# Anticancer and Anti‐Inflammatory Potential of Betalains: A Systematic Review on Preclinical Studies

**DOI:** 10.1002/fsn3.72133

**Published:** 2026-07-24

**Authors:** Aroob Fatima, Muhammad Tauseef Sultan, Farhang Hameed Awlqadr, Ahmad Mujtaba Noman, Hassan Raza, Zoha Saleem, Nimra Anees, Khaled Arab

**Affiliations:** ^1^ Department of Human Nutrition, Faculty of Food Science and Nutrition Bahauddin Zakariya University Multan Pakistan; ^2^ Food Science and Quality Control, Halabja Technical College Sulaimani Polytechnic University Sulaymaniyah Iraq; ^3^ Department of Food Science and Technology, Faculty of Food Science and Nutrition Bahauddin Zakariya University Multan Pakistan; ^4^ Department of Food Science and Technology, Faculty of Agriculture University of Tabriz Tabriz Iran

**Keywords:** anti‐inflammation, Bax, betacyanin, betalains, cancer, MAPK, TNF‐α

## Abstract

Betalains, water‐soluble pigments from beetroot and other Caryophyllales plants, have shown promising anticancer and anti‐inflammatory properties, though systematic evaluations remain limited. This review synthesized preclinical in vitro and in vivo evidence on betalains' bioactivity and mechanisms. Literature searches (2015–2025) across PubMed, ScienceDirect, Wiley Online Library, and Taylor & Francis identified 481 studies, with 25 meeting inclusion criteria under PRISMA guidelines. The risk of bias was assessed using multiple tools. The review has been registered in PROSPERO under ID 1172411. Findings indicate that betalains inhibit cancer cell viability and proliferation, including IC_50_ values of 64 to 107 μg/mL in colorectal cancer cell lines, induce apoptosis via caspase activation, increase G2/M phase arrest to 49.6% in A549 lung cancer cells, reduce oral squamous cell carcinoma viability from 89% to 50%, and regulate genes such as p53, Bax, and Bcl‐2. Anti‐inflammatory effects included suppression of NF‐κB signaling and reduction of TNF‐α, IL‐6, COX‐2, and iNOS, alongside enhanced antioxidant defenses. Risk of bias was generally low. Collectively, betalains emerge as safe, food‐derived compounds with potential for cancer and inflammation management. However, well‐designed clinical trials remain essential to establish efficacy and therapeutic applications in humans.

## Graphical abstract

Betalains derived from diverse natural sources, including beetroot (*Beta vulgaris*), *Opuntia ficus‐indica*, yellow prickly pear, and *Atriplex hortensis* “rubra” leaf and seed extracts, as well as purified betalains (amaranthin, celosianin, betanin, betaxanthins, and related derivatives), exhibit significant anticancer and anti‐inflammatory activities in preclinical models. These compounds inhibit cancer cell growth, induce apoptosis and cell cycle arrest, suppress pro‐inflammatory mediators and NF‐κB signaling, and enhance antioxidant defenses, highlighting their potential therapeutic roles in cancer prevention and inflammation reduction.

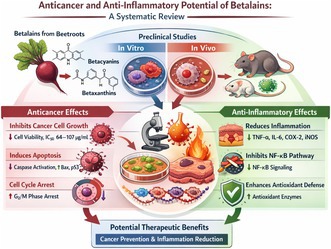

**Abbreviations**
BC: betacyanin
BHE: beetroot hydroethanolic extract
BX: betaxanthin
CRC: colorectal cancer
DAPI: 4′6‐Diamidino‐2‐phenylindole
FAS: Fas cell surface death receptor
GSH: cellular glutathione
HDAC: histone deacetylases
HO‐1: heme oxygenase‐1
MDR: multidrug resistance
NPs: nanoparticles
PARP: poly (ADP‐ribose) polymerase
UVA: ultraviolet A
UVB: ultraviolet B
UVR: ultraviolet radiation
XVX: vitexin‐2‐O‐xyloside

## 1 Introduction

Cancer is the second leading cause of death, affecting millions of people worldwide. According to statistics, approximately 10 million deaths due to cancer were registered in 2020, which means that one out of six deaths is related to this disease (World Health Organization, n.d.). Cancer is characterized by the mutation of normal cells into abnormal cells, which not only alters the surrounding tissues but also disseminates to distant sites in the body (Brown et al. 2023). The disruption of normal cellular processes due to lifestyle practices, environmental factors, and epigenetic mutations contributes to the development of malignancy (Nenclares and Harrington 2020). Cancer is also linked with inflammation in the body; an increase in inflammation, such as intestinal inflammation, increases the risk of gastric cancer risk (Ullman and Itzkowitz 2011). Inflammation is a defense mechanism of the body that involves vascular, immune, and cellular processes, which can exacerbate serious health conditions and must be reduced to slow the progression of diseases (Xing and Lin 2025). While there have been recent advancements in different disease management methods, such as cancer, treatments remain challenging due to high cost, drug resistance, and adverse side effects (Sterner and Sterner 2021). Therefore, there is a critical need for affordable, safe, and effective therapies to reduce this burden (Sung et al. 2021).

**FIGURE 1:**
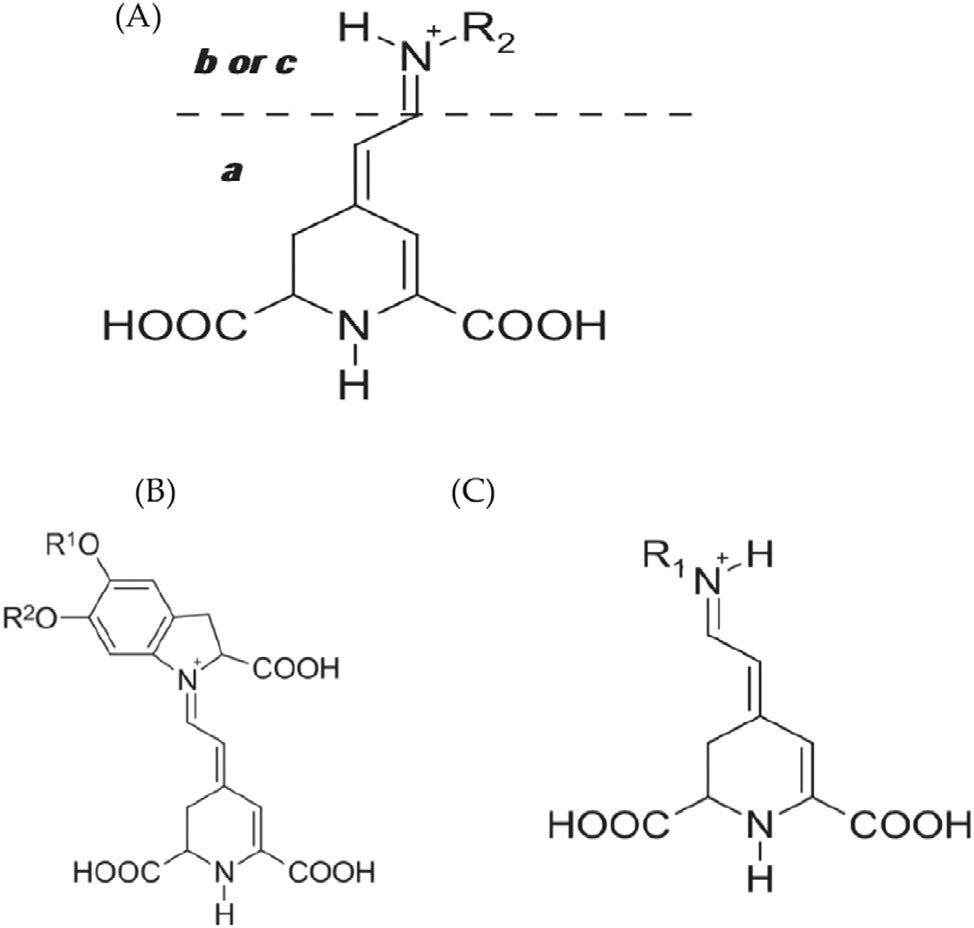
(A) The structure of the basic betalain formula: (a) betalamic acid moiety of all molecules; (b) *R1*‐N‐*R2* = residue of cyclo‐DOPA or cyclized stizolobinic acid (*Amanita muscaria*) in betacyanins; (c) *R1*‐N‐*R2* = residue of an amino acid or amine in betaxanthins. (B) The chemical structure of betacyanins: Betanidin: *R1* and *R2* = OH; Betanin: *R1* = glucose *R2* = OHRS. (C) The chemical structure of betaxanthins: *R1* = amino acid or amine; Indicaxanthin: Proline‐betaxanthin; Vulgaxanthin: Glutamine‐betaxanthin. Drawn using Marvin JS (ChemAxon n.d.).

Owing to the side effects and limitations of conventional therapy treatments, nonconventional therapies and plant‐based interventions are gaining global attention. Many researchers are exploring natural compounds with cost‐effective anticancer and anti‐inflammatory effects. Phytochemicals are plant‐sourced compounds with a reputation for chemopreventive and anti‐inflammatory properties (Marrero et al. 2022). Recently, scientists have discovered a promising role of phytochemicals from the Cactaceae family and their metabolites, which have been used as traditional medicines for centuries, in combating cancer (Orozco‐Barocio et al. 2024). Betalains, a group of water‐soluble, nitrogen‐containing pigments with the general structure shown in Figure 1A, drawn using Marvin JS (ChemAxon), are found in the Caryophyllales order (Miguel 2018). They are classified into two main types based on color: betacyanins, which have pigmentation ranging from red to purple or violet, such as betanin (found in beetroot), and betaxanthins, which produce colors ranging from yellow to orange (Kumorkiewicz‐Jamro et al. 2021). The color difference between betacyanins and betaxanthins is mainly due to the variations in their chemical structures. Betaxanthins typically appear yellow to orange due to the presence of a specific amino acid (Esquivel 2024). Figure 1B,C demonstrates the structural differences between betaxanthins and betacyanins.

Betalain has been used as an anti‐inflammatory agent and has chemopreventive potential against various cancer types. In cell lines and animal studies, betalains and their derivatives have been shown to reduce inflammation, downregulate inflammatory markers, trigger cell cycle arrest, enhance apoptosis, and regulate key signaling pathways related to the survival and growth of cancer cells (Yin et al. 2021). Despite these key findings and evidence, the data remain inconsistent and scattered for the anticancer and anti‐inflammatory experimental models. While betalains, particularly those from beetroot, have been widely studied for their biological activities, existing reviews often lack methodological rigor. This systematic review aims to address the research gap by being the first to systematically apply the PRISMA 2020 guidelines and the SYRCLE risk‐of‐bias techniques to studies published between 2015 and 2025. This approach was used to analyze and critically assess the existing evidence‐based data on the anticancer and anti‐inflammatory effects of betalains and their derivatives available on search engines such as PubMed, ScienceDirect, Wiley Online Library, and Taylor and Francis. The primary objective of this systematic review was to clarify the chemopreventive value of betalains in cancer and inflammation, and to identify research gaps and limitations for future studies.

## 2 Methodology

### 2.1 Reporting and Protocol

This systematic review adhered to the PRISMA (Preferred Reporting Items for Systematic Reviews and Meta‐Analyses) guidelines (Page et al. 2021). The completed PRISMA checklist 2020 is provided in Table S1. The PICO framework (Population/Patient, Intervention, Comparison, and Outcome) was redesigned based on this review and used to investigate the eligibility criteria and data collection for the systematic review (Methley et al. 2014). This systematic review protocol has been registered in PROSPERO with ID: 1172411.

#### 2.1.1 Population

The population in the review consisted of cancer cell lines from various cancer types derived from human sources, cell lines related to inflammation, and animal models of cancer and inflammation. Normal cells and animal‐sourced cell lines were also included (Fernando et al. 2023). Some of the included cell lines in the review were SSC‐4 and SCC131 (Zou et al. 2022), HT‐29 and Caco‐2 (Saber et al. 2023; Farabegoli et al. 2017; Wang et al. 2022), MCF‐7 (Nowacki et al. 2015), A549 (Chandrasekaran et al. 2022; Hirad et al. 2025), PA‐1 (Shafreen and Kumar 2022), MG‐63 (Liu et al. 2023), HeLa, T24 (Scarpa et al. 2016), HaCaT (Zand et al. 2024), and murine RAW 264.7 macrophages (Fernando et al. 2023). Some of the included animal models were *Caenorhabditis elegans* tumoral gonads (Henarejos‐Escudero et al. 2023) and male Sprague–Dawley rats (Tan et al. 2015).

#### 2.1.2 Intervention

The intervention involved the administration of betalains (betanin, betacyanin, and betaxanthin) to both cell lines and animal models. Betalains in pure form, incorporated as nanoparticles, or as a component of beetroot extract, isolated from other compounds, were used in this study. Studies investigating the isolated effects of betalains, including those with varying dosages, durations, and solvent types, were also included. In contrast, studies on betalains in combination with other anticancer agents (such as chemotherapeutic drugs or other bioactive compounds) were excluded. Betalain‐enriched plant extracts (such as beetroot extracts) or betalain‐enriched transgenic fruits and vegetables at different doses and concentrations are included in the review.

#### 2.1.3 Comparator

The comparators in the included studies consisted of untreated control cells and healthy cells. In untreated control cells, betalains (betanin or betacyanin) were absent, whereas all other conditions remained the same as those of treated cells (with betalains). Normal human cells without tumor growth were treated with betalains and compared to cancerous cells under the same treatment. Vehicle groups treated with distilled water or DMSO were used as comparators in animal models.

#### 2.1.4 Outcomes

The outcomes revealed different mechanisms of action of betalains against cancer. Reduced inflammation, modulation of cancer‐related gene expression (Bax, Bcl‐2), and inflammatory markers (such as IL‐6, COX‐2, IL‐1β, TNF‐α, and iNOS); promotion of apoptosis, validated by DNA damage (fragmentation) increased Caspase‐3 and Caspase‐8 activity levels; reactive oxygen species (ROS) generation and mitochondrial dysfunction; and inhibition of cell viability and proliferation, as observed through MTT or SRB assays. Furthermore, the PI3K/Akt/mTOR, MAPK, and NF‐κB signaling pathways were also influenced by betalain treatment, indicating the anticancer and anti‐inflammatory potential of betalains.

### 2.2 Search Strategy

An exhaustive search of PubMed, ScienceDirect, Wiley Online Library, and Taylor & Francis was performed on November 27, 2025, to collect data from 2015 to 2025. Additionally, the snowballing method was applied to identify related articles by examining the reference lists of the included studies for additional eligible sources. To maintain the comprehensiveness of the review, gray literature (including conference proceedings, theses, and unpublished studies) was searched using search strings that combined Medical Subject Headings (MeSH) terms and keywords using Boolean operators (AND, OR). The terms for bioactive compounds (“betalain,” OR “betanin,” OR “betacyanin”) with cancer indicators (“betalain AND cancer,” OR “betalain AND tumor,” OR “inflammation,” OR “inflammatory markers”) and in vitro/in vivo indicators (“cell line,” OR “betalains AND animal model,” OR “rat modeling”). Studies that met the inclusion criteria and that minimized publication bias, such as articles published in English only, were included in the analysis. Unpublished articles, reviews, editorials, articles in languages other than English, and conference abstracts were excluded from the search.

### 2.3 Eligibility Criteria

Distinct and clear eligibility criteria were established to prepare this review. The main goal was to identify preclinical (in vitro and in vivo) studies on the effect of betalains on cancer and inflammation. Detailed inclusion and exclusion criteria were determined to ensure the selection of relevant studies.

#### 2.3.1 Inclusion Criteria

The inclusion criteria based on the PICOS framework are as follows: (i) population: human cell lines (such as Caco‐2, HeLa, MCF‐7, and A549) of all cancer types/other cell lines and animal models of any species (such as Wistar rats, Syrian hamsters, and *C. elegans* tumoral gonads); and (ii) Intervention and Comparators: betalains and their derivatives, irrespective of dose and duration of intervention, were eligible for inclusion. The group treated with bioactive compounds, compared with the nontreated group (negative control), was also included; (iii) outcome measured: anticancer and cytotoxic effects on the cancer cells of the studied compound, as observed through cell viability, apoptosis, tumor size and histological changes, protein and gene expression, reduced inflammation, and analysis of molecular targets and signaling pathways linked with their anticancer and anti‐inflammatory activity; and (iv) study design: controlled in vitro studies involving human cell lines and in vivo studies for cancer and inflammation.

#### 2.3.2 Exclusion Criteria

The following studies were excluded from this systematic review: (i) studies that used in silico analysis only; (ii) reviews, editorials, or meta‐analyses; (iii) studies not involving betalains as a single bioactive compound; (iv) studies using betalain or its derivatives in combination with other anticancer treatments, such as radiotherapy or agents/drugs, such as cisplatin; (v) studies in languages other than English; and (vi) studies involving human subjects. Two reviewers independently screened the articles, and any disagreements were resolved by consensus.

#### 2.3.3 Screening of Articles

The screening process followed two stages: a preliminary review of titles and abstracts, followed by full‐text screening of potentially eligible studies. Of the 481 initially identified records, 52 were excluded. During the title and abstract screening phase, 293 studies were excluded. These exclusions were due to inappropriate study designs (e.g., editorials or reviews) and to irrelevant studies identified from titles and abstracts. After these exclusions, 136 studies remained, of which 97 could not be fully accessed initially despite extensive efforts to use alternative methods, search institutional repositories, and contact authors; 80 studies were retrieved in the end, while 17 remained unable to be accessed. Of these 80 studies, 76 were excluded, and only 4 were included in the end. These excluded studies were listed in detail in Appendix S1 to ensure transparency. The table presents the names of the excluded studies, the reasons for their exclusion, and pertinent information that enables easy comprehension of how the selection was conducted and which studies were not included in the review. Ultimately, the full texts of 119 articles were screened for eligibility. After screening for eligibility, 94 studies were excluded (out of which 76 were from retrieved data); of those, 19 were excluded for studies with in silico or human study design, 18 were removed due to betalains combined with other anticancer agents such as chemotherapeutic drugs or bioactive compounds like gingerol or quercetin or drug like doxorubicin, 33 studies were excluded based on irrelevant outcome, 21 studies were removed as they observed effects (disease) other than anticancer and anti‐inflammatory (such as antioxidative effect only), 2 studies for incomplete methodology, and one was removed because of language other than English. Only 25 studies were included in this systematic review.

### 2.4 Risk of Bias

The risk of bias was assessed using multiple tools due to the heterogeneity of the data. Because there is no standardized tool to evaluate risk of bias (RoB) in in vitro studies, a customized tool was designed for both cancer‐ and inflammation‐related studies. The tool used in this review was adapted from Abdull Rahim et al. (2024). The customized RoB tool consisted of seven different domains: (i) cancer cell lines involved in the study, (ii) duration of intervention/exposure to the cancer cell culture, (iii) concentration used in the cancer cell culture, (iv) cell culture medium used for control, (v) tools for analyzing the outcome/result, (vi) triplication of experiments, and (vii) the number of independent experiments conducted. Each domain was indicated by a “yes,” “no,” or “unclear” response. A study with a score of > 80% or < 50% “yes” was judged to have low or high risk of bias, respectively. A moderate risk of bias was considered for any score between these percentages, as shown in Table S2. For the inflammation‐related in vitro studies (*n* = 05), the Quality Assessment Tool for In Vitro Studies (QUIN) was used to evaluate the studies with “low” used for adequately specified data with less risk of bias, “unclear” for inadequately specified, and “high” for not specified, higher risk of bias in the study (Ridho et al. 2024). Overall risk criteria include studies with 4 or fewer low‐risk of bias were considered as “high risk,” and 6 or more low RoB were regarded as “low risk”; the in‐between values were labeled as “moderate risk” in the overall section, as mentioned in Table S3.1. The domains related to the QUIN Tool were described in Table S3.2. The traffic plot of all in vitro studies was developed using the visualization (Robvis) tool (McGuinness and Higgins 2021).

To assess the RoB of in vivo studies, the SYRCLE RoB tool (Systematic Review Center for Laboratory Animal Experimentation) was used, following the method used by Ruivo et al. (2024). The presence of data represented “low risk” while the absence of data represented “high risk” along with inadequate information, which was considered “unclear” as presented in Appendix S2. The domains were discussed in Appendix S3. The heatmap and the stacked bar chart summary for the in vivo studies were created in an Excel spreadsheet. The risk of bias for each included study was independently assessed by two reviewers. In case of disagreements, the reviewers discussed the discrepancies until a consensus was reached. If a consensus could not be reached, a third reviewer was consulted to make the final decision. This approach ensured that the risk of bias was thoroughly evaluated and that reviewers agreed.

### 2.5 Data Extraction

Studies were identified based on titles and abstracts after extracting articles published from January 2015 to May 2025. This ten‐year time window was selected to focus on recent studies that reflect the latest findings in betalain research. Although this time frame was carefully selected to ensure up‐to‐date information, it may exclude certain mechanistic studies from the early 2010s that could have added value to the review. The implications of this limitation are discussed in the limitations section. To determine the final study selection, the full texts of all selected papers were examined independently. Duplication studies were identified and excluded. The following data were compiled from the screened studies: author and date of study, country, primary focus of study (e.g., cancer type, inflammation), population (cell lines and animal model), intervention and their durations, comparators, types of assays used for in vitro analysis, and main outcomes/findings, as mentioned in Table 1 for the included in vitro studies and Table 2 for included in vivo studies.

**TABLE 1:** Descriptive characteristics of included in vitro studies (*n* = 17).

References (country)	Primary focus of study	Population/cell lines	Intervention/treatment	Purity of betalain used	Duration	Comparator	Assays	Key findings
Zou et al. (2022) (China)	Oral cancer	SSC4, SCC131	5–70 μM Betanin	(Analytical grade)	24 h	Untreated control OSCC and SCC4 cells	MTT assay	↓ Cell viability ↓ *Bcl‐2 mRNA* expression ↑ *Bax* ↑ caspase‐3 ↑ caspase‐9 ↓ PI3K/AKT/NF‐κB
Saber et al. (2023) (Iran)	Colorectal cancer	Caco‐2, HT‐29	20 to 140 μg/mL Betanin extracted from beetroot	Betanin comprises 75%–95% of red beetroot pigments	24–48 h	Caco‐2 and HT‐29 cells without betanin treatment, 293 normal kidney cells	MTT assay	↑ *BAD* ↑ Caspase‐3 ↑ Caspase‐9 ↑ Caspase‐8 ↑ *Fas‐R*
Shafreen and Kumar (2022) (India)	Ovarian cancer	PA‐1	40 μg/mL Betanin	75%–90% of the total pigment	24 h	Control cell (no betanin treatment)	MTT assay	↑ *p53* ↑ *Bax* ↑ Apoptosis ↓ Membrane potential.
Liu et al. (2023) (China)	Osteosarcoma	MG‐63	27.24 and 36.33 μM Betanin	Betanin (purity 97%)	24 h	L929 normal fibroblasts	Cell viability assay Comet assay	↓ MG‐63 cell growth ↓ PI3K ↓ AKT ↓ S6 ↓ mTOR
Nowacki et al. (2015) (France)	Breast cancer	B16F10, MCF‐7, MDA‐MB‐231, and HT‐29	30 μM Betanin/Isobetanin	80% purity, with betanin comprising 64% and isobetanin comprising 36% of the total betalains.	48 h	HUVEC, MRC‐5 normal cells with betanin treatment	Proliferation assay Autophagy assay	↑ *BAD* ↑ *TRAILR4* ↑ *FAS* ↑ *p53* ↑ Intrinsic and extrinsic apoptotic pathways.
Chandrasekaran et al. (2022) (India)	Lung cancer	A549	73.5 μM/mL Betanin	(Analytical grade)	24 h	Control cells (A549 cells treated with DMSO alone)	Cytotoxicity assay	↑ Cell death ↑ *BcL‐2* ↑ Caspase‐3 ↑ *CDK‐6* ↑ *Topoisomer‐* *ase I and II proteins*.
Scarpa et al. (2016) (Italy)	Bladder cancer	T24	50 μg/mL Betacyanin	Purity not mentioned	24 h	NCTC 2544 normal human skin keratinocytes	SRB assay FACS analysis	↑ Caspase‐8 ↑ Caspase‐3 ↑ *Bax* ↓ *BIRC5* (*survivin*) ↓ *CTNNB1* (*β‐Catenin*)
Zand et al. (2024) (Hungary)	Skin cancer	HaCaT	20 to 80 μM Betanin	Purity not mentioned	24 h	HaCaT cells without betanin or UV exposure	Comet Assay Cell Viability/XTT Assay	↓ *DNMT1* ↓ *DNMT3A* ↓ *DNMT3B* ↓ *HDAC5* ↓ *HDAC6*
Hirad et al. (2025) (Saudi Arabia)	Lung cancer	A549	100, 125, and 150 μg/mL Betanin‐ZnO NPs	Purity not mentioned	48 h	A549 without Betanin ‐ZnO NPs	Cytotoxicity assay Trypan blue exclusion assay Superoxide dismutase assay	↑ *NO* ↑ *GSH* ↑ SOD ↑ CAT ↑ *Bax* ↑ p53 ↓ *Bcl‐2* ↓ MMP
Farabegoli et al. (2017) (Italy)	Colon cancer	Caco‐2	0.35 μg/mL Betaxanthin (BX) and 0.25 μg/mL Betacyanin (BC)	Purity not mentioned	24, 48, and 72 h	Untreated Caco‐2 cells	SRB assay (Sulforhodamine B), ROS assay	↓ *COX‐2* ↓ *IL‐8* ↓ *Bcl‐2* ↑ *Bax* ↑ Caspase‐3
Yin et al. (2021) (China)	Nonsmall cell lung cancer (NSCLC)	A549, 16HBE (normal human bronchial epithelial cells)	Betalain	Purity not mentioned	10 to 50 μM for 48 h	Untreated control cells	MTT assay, flow cytometry, LDH cytotoxicity detection kit, FITC/PI assay kit	↓ p‐PI3K, p‐Akt, and mTOR levels (suppressing the PI3K/Akt/mTOR signaling pathway) ↑ p53 and p21 expression ↑ apoptotic cells of A549 cells.
Smeriglio et al. (2021) (Italy)	Intestinal Inflammation	Human intestinal epithelial cell line (Caco‐2)	62.50–500 μg/mL and 125–1000 μg/mL for betanin, Indicaxanthin ( *Opuntia ficus‐indica* )	Purity not mentioned	30 min for BSA denaturation assay	Treatment with pure betalains and prickly pear extract against untreated cells	MTT assay, FRAP assay, BSA denaturation assay	↓ ROS level ↓ Secretion of inflammatory mediators (*IL‐6, IL‐8, NO*) ↓ NF‐κB signaling pathway
Kumorkiewicz‐Jamro et al. (2025) Iran	Cytotoxic activity and apoptosis‐inducing mechanisms of betanin against U87MG human glioma cells	Human‐derived cell lines (U87MG glioma cells) and normal human lymphocytes	0, 1, 3, 7, and 14 mg/mL Betanin, a betalain pigment from beetroot ( *Beta vulgaris* )	Purity not mentioned	12 h	Normal human lymphocytes acted as noncancerous controls	MTT assay, Mitochondrial Function Assays	↓ Cell viability ↑ Caspase‐3 activity ↓ SDH activity ↑ Cytochrome c release ↑ ROS formation
Fernando et al. (2023) (United Kingdom)	Inflammatory and oxidative stress on murine macrophages	Murine RAW 264.7 macrophages	Betacyanins: Betanin and Neobetanin, Betaxanthins: Indicaxanthin and Vulgaxanthin I (Red beetroot, yellow prickly pear) 1 to 100 μM	Betanin (BET): 97% Neobetanin (NEO): 52% Vulgaxanthin I (VGX): 79% Indicaxanthin (IDX): 95%	1 to 24 h	positive control‐ 10 μM curcumin	RNA isolation and quantitative real‐time PCR, EPR	↓ *NOX‐2* mRNA levels ↑ *IL‐10* mRNA levels ↓ Pro‐inflammatory markers (*IL‐6*, *IL‐1β*, and *iNOS*)
Wang et al. (2022) (United Kingdom)	Inflammatory and oxidative stress on intestinal epithelial cell line (Caco‐2)	Human intestinal epithelial cell line (Caco‐2)	Betanin, Vulgaxanthin I ( *Beta vulgaris* L.), Indicaxanthin ( *Opuntia ficus‐indica* L.) at 5–80 μM betalains	Betanin: 97% purity Vulgaxanthin I: 79% purity Indicaxanthin: 95% purity	6 h	Cytokine‐stimulated cells without treatment, a positive control like 10 μM curcumin, for the anti‐inflammatory effect	Cellular uptake analysis, ROS measurement, and intracellular radical scavenging via EPR (Electron Paramagnetic Resonance)	↓ Pro‐inflammatory cytokine gene expression ↑ Antioxidant defenses, free radical scavenging
Salimi et al. (2021) (Australia)	Antioxidant and anti‐inflammatory properties of *Atriplex hortensis* “*rubra*” extracts and betalain pigments	RAW264.7 (murine macrophage), THP‐1 (human leukemia monocytic)	*A. hortensis* “rubra” leaf and seed extracts, purified betalains (amaranthin, celosianin), LPS (lipopolysaccharide)	Purified betalains (amaranthin and celosianin)	1–2 h pre‐treatment, LPS stimulation 16–24 h	Trolox (standard), positive control for Luciferase assay	PGE2 ELISA, Griess Reagent Nitrite Measurement, Western blot, Luciferase assay, CAA assay, DI‐IM‐MS, LC‐DAD‐ESI‐MS/MS	**↓** IL‐6 and IL‐1β secretion in LPS‐stimulated RAW264.7 cells **↓** NF‐κB promoter activity **↓** Nos2 AND Cox2 protein levels
Rusak et al. (2024) (Poland)	Cytotoxicity and anti‐inflammatory effects of gomphrenin‐rich fraction and crude extract from *Basella alba L. f. rubra* fruits	NHDFs, L929 cells, THP‐1 cells	Gomphrenin‐enriched fraction (A), unpurified crude extract (B); concentrations from 0.001 to 40 μg/mL	Purity not mentioned	1 h for ROS, 1–24 h for LPS, 30 min for Griess and MDA assays	Positive control: H_2_O_2_ for ROS; LPS for inflammation; Meloxicam and Indomethacin for anti‐inflammatory activity	SRB assay, DCF‐DA assay, Griess assay, MDA assay, ELISA, NF‐κB, and COX activity assays	**↓** IL‐1β and IL‐6 cytokines ↓ ROS, NO, and lipid peroxidation ↓ NF‐κB and COX activity

**TABLE 2:** Characteristics summary of included in vivo studies (*n* = 09).

References (country)	Primary focus of study	Population/animal	Bioactive compound/source/purity of betalain used	Intervention	Comparator	Outcome
Yin et al. (2021) (China)	NSCLC	Athymic male BALB/c nude mice	Betalain (Analytical grade)	25 and 50 μM via intraperitoneal injection (IP) for 4 weeks	0.1% DMSO in mice (control group)	↓ Tumor Volume and Weight ↓ Xenobiotic Dysfunction Marker Enzymes ↓ Serum Tumor Marker (*CEA*) and ↓ Pro‐inflammatory Cytokines (*TNF‐α, IL‐6, IL‐1β*) ↑ apoptotic cells
Duraisamy et al. (2025) (India)	7, 12‐ Dimetylbez[a]anthracene (DMBA) Oral squamous cell carcinoma (OSCC)	36 Male Syrian golden hamsters aged 8–10 weeks (80 and 120 g)	Betanin (purity not mentioned)	10, 20, and 40 mg/kg for 14 weeks via oral gavage	Untreated group, 0.5% DMBA for 10 weeks	↓ Phase I enzymes (*Cyt P450, b5*) and ↑ Phase II enzymes (*GST, GR, GSH, DTD*) ↓ LPO by‐products (*TBARS, LOOH, CD*) ↑ levels of antioxidants
Henarejos‐Escudero et al. (2020) (Spain)	*C. elegans* tumor model	TJ375, JK1466 ( *C. elegans* mutant strain)	Pure betalains: tryptophan‐betaxanthin, betanin, indicaxanthin, phenylethylamine‐ and phenylalanine‐betaxanthin (purity not mentioned)	25 μM for several days via oral feeding	Untreated or vehicle group	↓ Oxidative Stress ↓ Tumor Size ↑ Lifespan by 9.3% and 11.4%
Henarejos‐Escudero et al. (2023) (Spain)	*C. elegans* tumoral gonads	JK1466 tumoral gonads model strain with gld‐1 knockdown	Tryptophan‐betaxanthin and derivatives (purity not mentioned)	25 μM for 2 days via soaking, absorbing by worms	Vehicle group	↓ Gene Expression (*rict‐1* and *daf‐15*) ↓ genes involved in cell growth ↓ Tumor Size
Tan et al. (2015) (China)	Inflammation and oxidative stress in paraquat‐induced acute kidney injury	40 male Sprague–Dawley rats (220 ± 20 g)	Betanin (purity not mentioned)	25 and 100 mg/kg/day for 5 days via intra‐gastric gavage	Vehicle group (no paraquat, distilled water), Untreated group	↓ *iNOS* and *COX‐2* protein levels, ↓ *NF‐κB* ↑ *SOD* and *CAT* (antioxidant enzymes) ↑ in kidney/body weight ratio
Yang et al. (2016) (China)	Isoproterenol‐induced acute myocardial infarction (AMI) with inflammation and oxidative stress	Adult male Sprague–Dawley rats (260–300 g)	Betanin (purity not mentioned)	25 and 100 mg/kg/day for 3 days via subcutaneous injection	Control group (normal rats with saline), AMI group with saline only	↓ NF‐κB protein expression (in rats with isoproterenol‐induced AMI) ↓ iNOS protein expression ↓ Inflammatory response in myocardial injury
Albasher et al. (2019) (Saudi Arabia)	Inflammation, apoptosis, and oxidative stress in CPF (chlorpyrifos)‐induced liver injury	11 weeks old, 28 adult Wistar rats (140–160 g)	Red beetroot extract (RBR) (Purity not mentioned)	300 mg/kg for 28 days via oral gavage	Control group (distilled water), CPF group (10 mg/kg CPF in corn oil)	↓ serum levels of *ALT, AST, ALP*, and bilirubin ↑ *Bcl‐2*, ↓ *Bax* ↓ Pro‐Inflammatory Cytokines
Saito et al. (2023) (Japan)	Inflammation in Colitis IBD (Inflammatory Bowel Disease)	7‐week‐old female C57BL/6 mice (18‐20 g)	Betanin and isobetanin in transgenic tomato and potato extracts (purity not mentioned)	164 and 239 μM betanin, 22 and 20 μM isobetanin for 11 days daily	Control group (treated with distilled water)	↓ body weight loss ↓ disease activity index (DAI) ↓ pro‐inflammatory gene transcripts (*Tnf, Il6, Ptgs2*).
Martinez et al. (2020) Brazil	Analgesic and anti‐inflammatory effects of betalain‐rich dye	Male Swiss or C57BL/6 strains	Betalain‐rich dye extracted from *Beta vulgaris* (beetroot) (purity not mentioned)	0–1000 mg/kg after 30 min of stimulus	Vehicle (saline) Indomethacin (Indo) at 10 mg/kg, used as a positive control for anti‐inflammatory effects	↓ cytokine levels in paw tissue ↓ oxidative stress markers ↓ cytokine production and oxidative stress in in vivo models

## 3 Results

### 3.1 Study Selection

**FIGURE 2:**
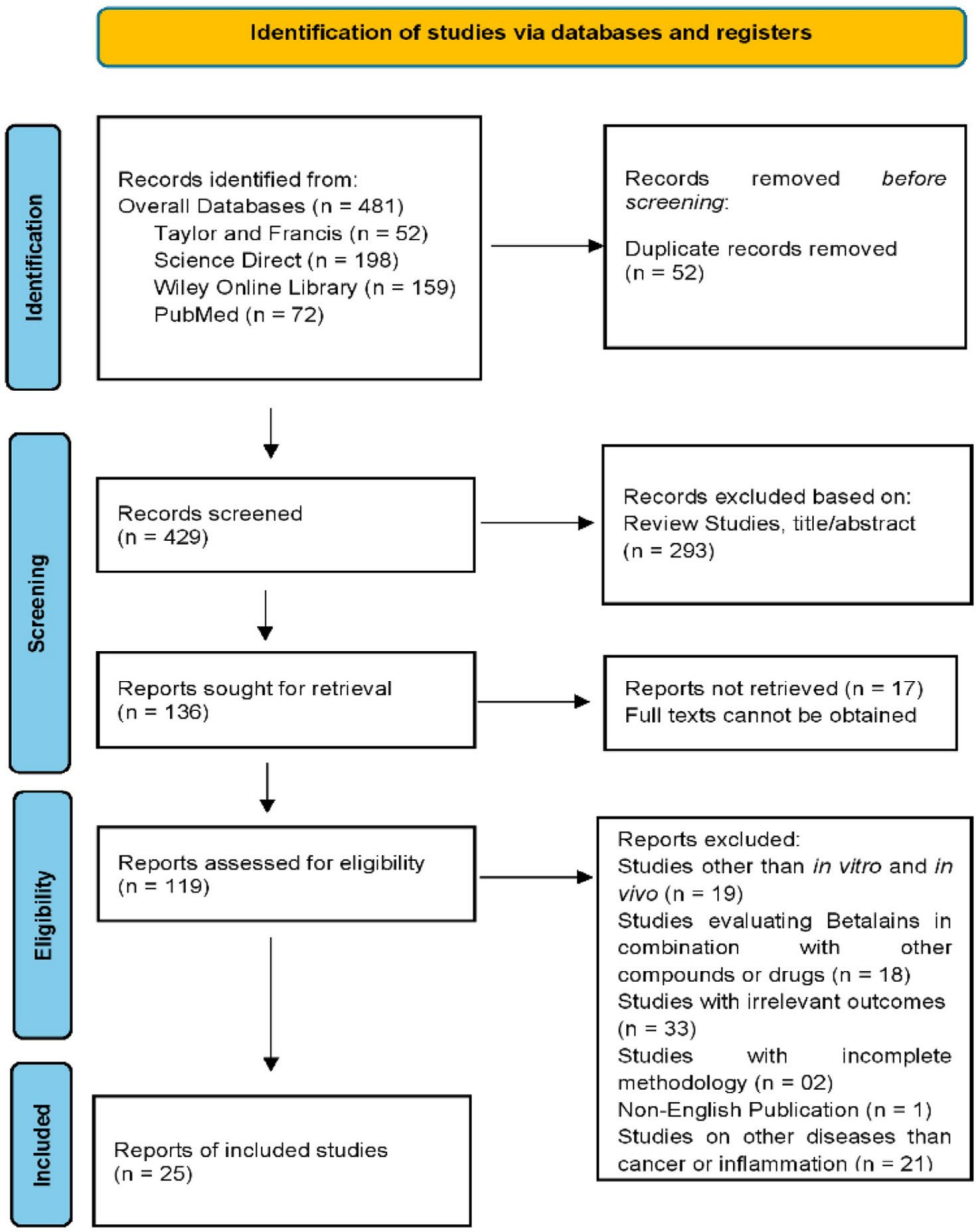
PRISMA flow diagram showing the selection, inclusion, and exclusion of the initially identified studies. This flowchart demonstrates how this systematic review selected the studies. A total of 481 records were identified across databases and registers, and after filtering for titles, abstracts, and eligibility criteria, 25 studies were included in the review. It is designed in accordance with the PRISMA 2020 guidelines, which ensure transparency and understanding of the study selection process (Page et al. 2021).

This systematic review was conducted in accordance with the PRISMA (Preferred Reporting Items for Systematic Reviews and Meta‐Analyses) guidelines, which are established standards for designing systematic reviews. Analysis of the databases identified 481 studies (Figure 2). After screening all abstracts of 481 studies and removing duplicates, 136 articles were identified for full‐text screening. The reviewers had a consistent and unambiguous agreement during the examination of the identified studies. Twenty‐five studies were eligible after all the selection criteria were met.

### 3.2 Study Characteristics

The selected studies were published between 2015 and 2025. Only articles published in English were included in this study. These articles originated from seven countries in various regions. The articles included in this systematic review were from around the world, including Asia, Europe, and the Middle East. Preclinical studies were included in this review. The dosage of betalain, treatment duration, cell lines, and animal models used in the selected studies varied. Multiple human cell lines were used in the in vitro studies, including Caco‐2, SSC‐4, SCC‐131, HT‐29, PA‐1, G‐63, MCF‐7, A549, T24, and HaCaT. Various animal species have also been used in in vivo studies. Of the 25 studies, nine were of the in vivo study type, 17 were of the in vitro study design only, whereas one study (Yin et al. 2021) was of both in vitro and in vivo study types. These features of the studies are presented in Tables 1 and 2.

### 3.3 Risk of Bias Assessment

**FIGURE 3:**
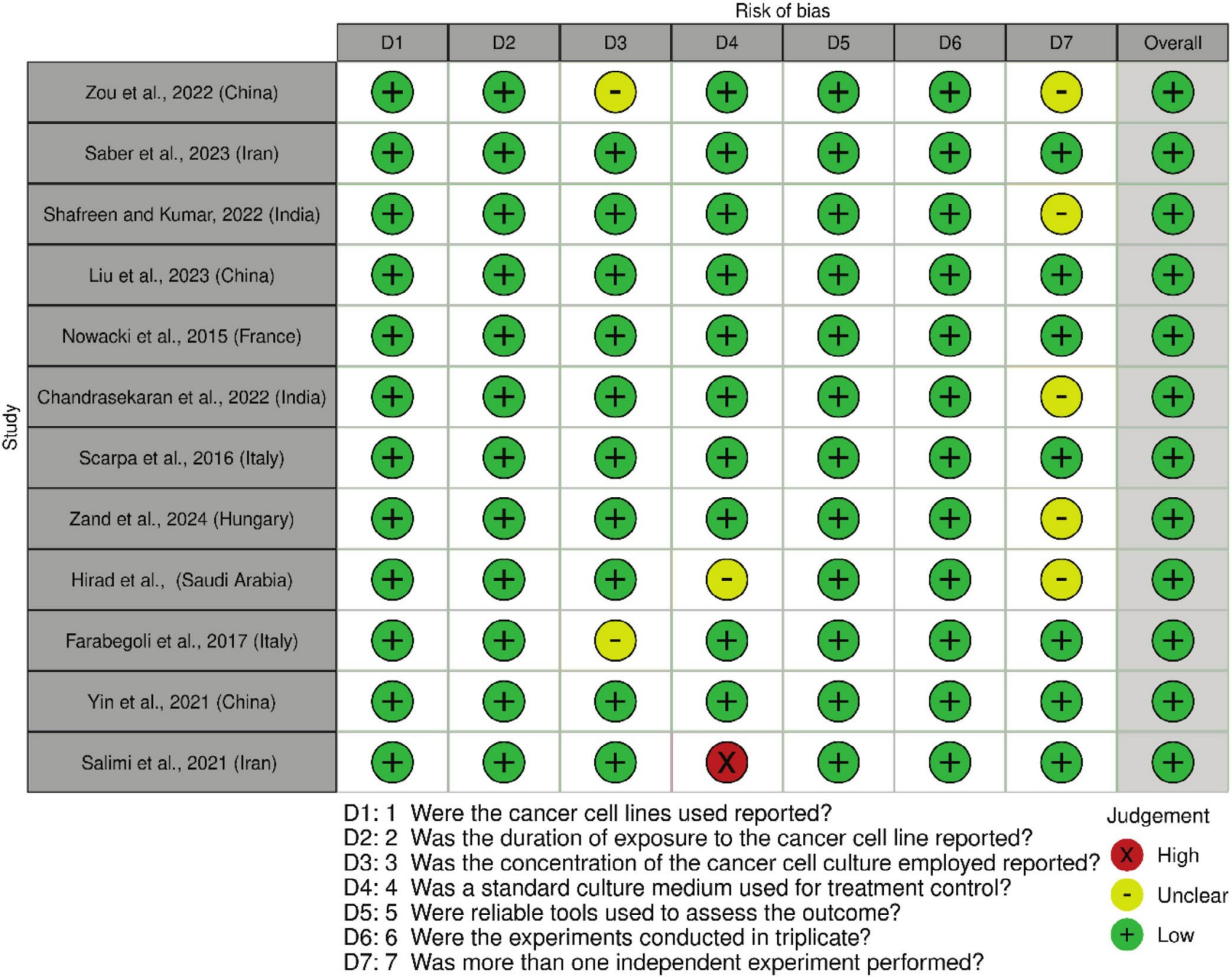
Traffic plot of customized RoB assessment of included cancer‐related in vitro studies (*n* = 12) (McGuinness and Higgins 2021). Green color represents low risk of bias, red represents high risk of bias, and yellow represents unclear risk of bias.

**FIGURE 4:**
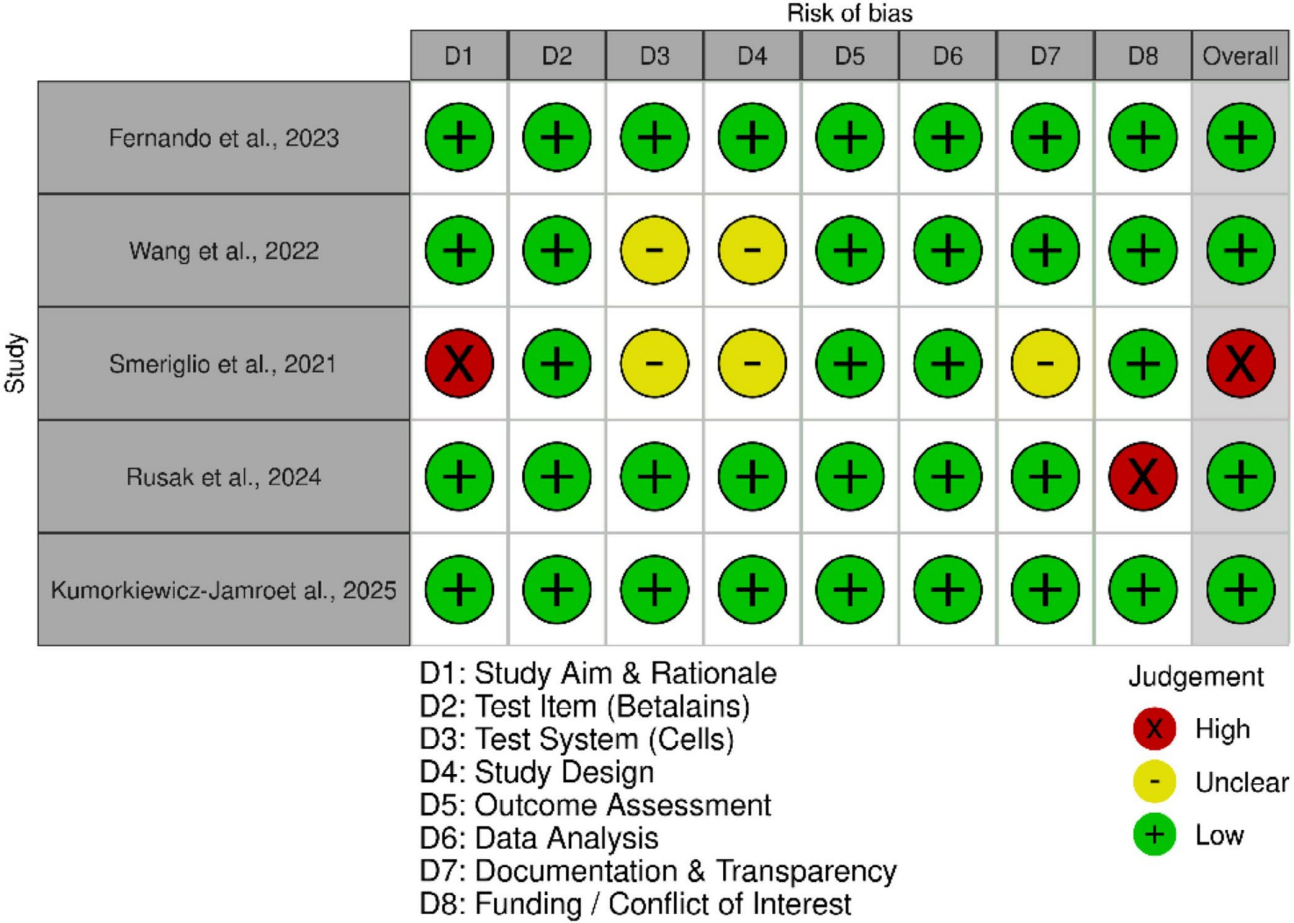
Traffic plot of RoB assessment of included inflammation‐related in vitro studies (*n* = 5) (McGuinness and Higgins 2021). Green color represents low risk of bias, red represents high risk of bias, and yellow represents unclear risk of bias.

**FIGURE 5:**
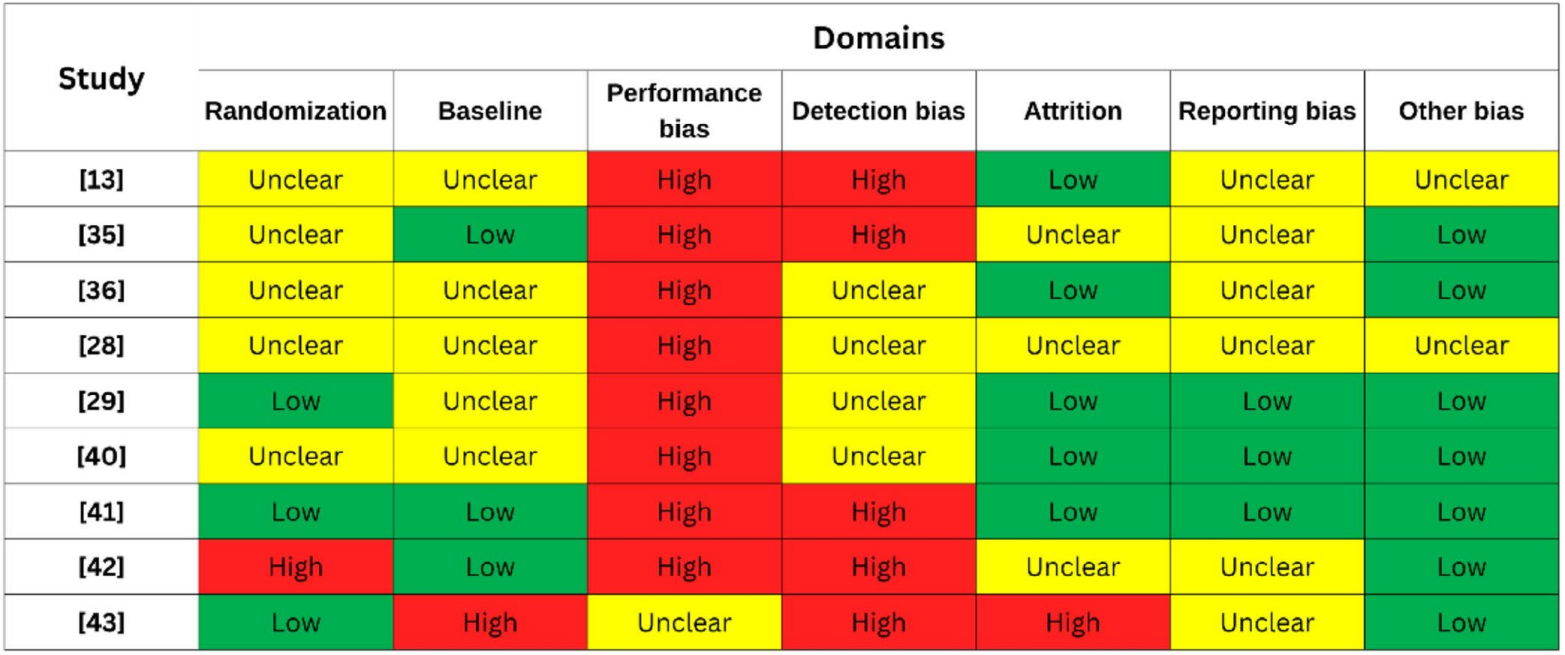
Heatmap plot of RoB assessment of included in vivo studies (*n* = 9). Green color represents low risk of bias, red represents high risk of bias, and yellow represents unclear risk of bias.

**FIGURE 6:**
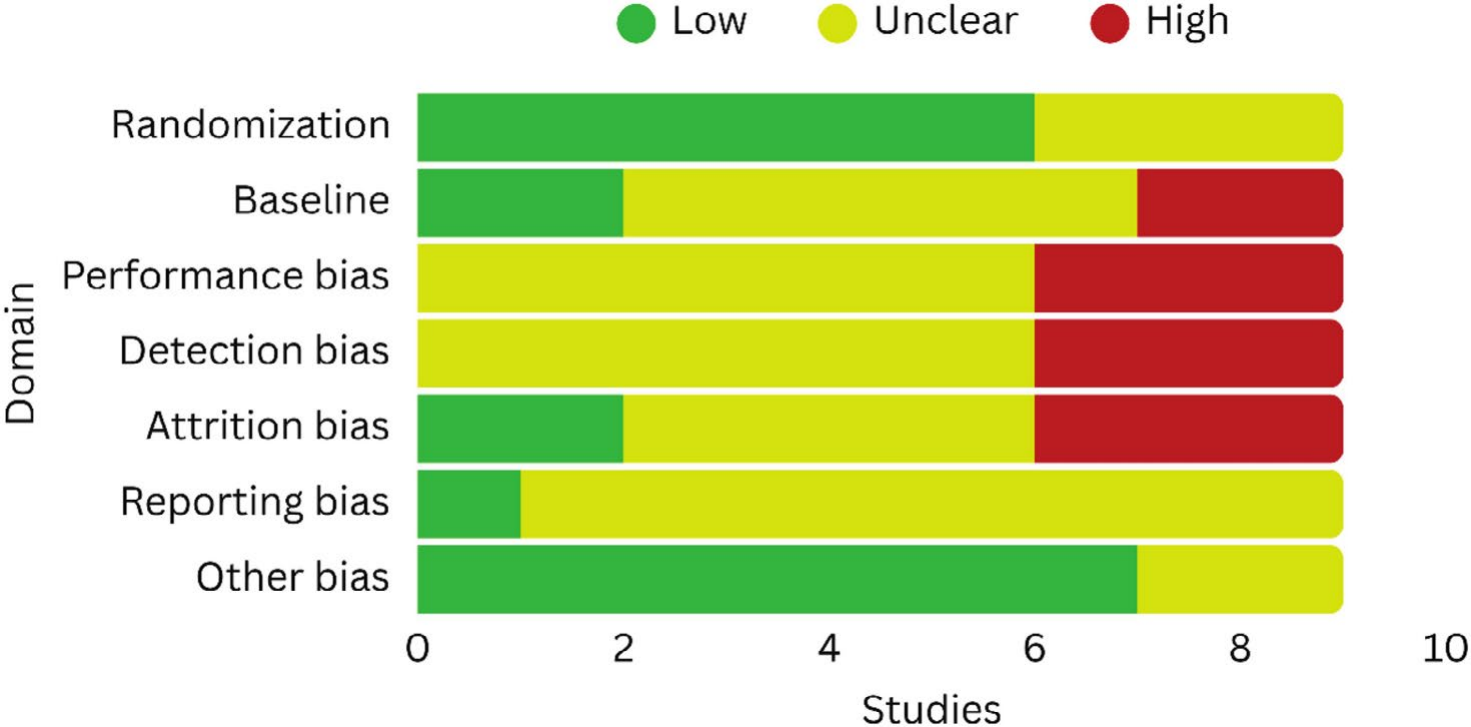
Summary stacked bar plot of RoB assessment of included in vivo studies per domain (*n* = 9). Green color represents low risk of bias, red represents high risk of bias, and yellow represents unclear risk of bias.

The risk of bias (RoB) assessment for cancer‐related in vitro studies is shown in Figure 3 and Table S2. The domains related to the type of cancer cell lines used, the duration of intervention/exposure, the tools used to assess the outcome, and the triplication of experiments were low risk in all the included studies. The concentration of cancer cell culture implementation was unclear in two studies, while the remaining studies (*n* = 10) in this domain were judged to be at low risk. The standard culture medium for the treatment control was unclear in only one study, and high risk of bias in another single study (Yang et al. 2016), whereas the remaining studies (*n* = 10) had a low risk of bias. At the same time, the number of independent experiments was unclear (*n* = 5) or low risk (*n* = 7). All 12 included studies were considered to have a low risk of bias. As for the 5 inflammation‐related in vitro studies, the overall risk was low, but one study had a high overall RoB, as shown in Figure 4. The in vivo studies were presented in two forms. At the individual study level, a heatmap showed that some domains, such as baseline and other biases, were occasionally at low risk, and that detection bias was mostly unclear across studies (Figure 5). The overall summary demonstrated that most studies were at a high risk of performance bias, while the risks of randomization and reporting bias were frequently unclear, as shown in Figure 6.

### 3.4 Inhibition of Cancer Cell Proliferation and Viability

Betalains have shown remarkable potential in combating cancer. Our systematic review illustrated that betalains inhibit cell proliferation and migration and disrupt the cell cycle at key stages. The included studies demonstrated an inhibitory effect on cell viability and proliferation across various cancer types. Betanin, a major component of betalains, displayed strong cytotoxicity against colorectal cancer (CRC) cell lines HT‐29 and Caco‐2 compared with normal KDR/293 cells, and no cytotoxicity was observed even at the highest tested concentration, validating betanin's selectivity for cancer cells (Saber et al. 2023). Another study on osteosarcoma showed a parallel effect: betanin selectively reduced MG‐63 cell growth in a dose‐dependent manner, with no cytotoxicity to normal L929 fibroblasts. It also notably downregulates MG‐63 cell migration (Liu et al. 2023; Rusak et al. 2024). Betanin has been shown to reduce the viability of SCC131 and SCC4 cells in oral squamous cell carcinoma (OSCC) from 89% to 50% (Zou et al. 2022). Betalains may reduce cancer cell viability; limited evidence from in vitro studies shows a reduction in U87MG glioma cell viability, with an IC50 of approximately 7 mg/mL. Yet this response required a relatively high concentration, and the use of a single cell line limits the generalizability. But this exhibits comparatively lower toxicity toward normal lymphocytes (Kumorkiewicz‐Jamro et al. 2025). Betanin (20 and 40 mg/kg) completely cured and protected against OSCC by inhibiting tumor incidence, volume, and burden, while improving body weight and maintaining normal histopathology compared to the DMBA group. It also normalized the antioxidant levels, lipid peroxidation, and detoxification enzyme activities, indicating a chemoprotective effect (Duraisamy et al. 2025). Morphological changes, including shrinkage, membrane blebbing, detachment, and shape distortion, were observed when PA‐1 ovarian cancer cells were treated with 40 μg/mL betanin (Shafreen and Kumar 2022). Betalains inhibited MCF‐7 breast cancer cells, in the form of betanin/isobetanin (Bet./IsoBet.), by decreasing cell viability and inducing morphological changes, such as reduced spreading and aggregation, in tumor cells (Nowacki et al. 2015). Similarly, Chandrasekaran et al. investigated the effects of betanin on the cell cycle of A549 lung cancer cells, suggesting that betanin exerts cytotoxic effects by promoting apoptosis, which leads to cell death. Cell cycle analysis revealed that betanin treatment reduced the percentage of cells in the G1 phase (14.2%) and slightly downregulated the S phase (30.5%), whereas the G2/M phase was strongly elevated (49.6%). Hirad et al. (Hirad et al. 2025) conducted experiments on A549 lung cancer cells using betalains in the form of Betanin‐coated zinc oxide nanoparticles (Betanin‐ZnO NPs) to enhance the stability and bioavailability of the compound, as it is sensitive to pH, temperature, and light. The results demonstrated inhibition of cancer cell proliferation, characterized by pronounced morphological changes, including shrinkage, blebbing, detachment, and a distorted appearance. Betalain significantly reduced A549 cell viability by modulating LDH release, inducing G2/M cell cycle arrest, and suppressing NSCLC tumor growth in vivo, as evidenced by reductions in tumor volume, weight, and serum CEA levels (Yin et al. 2021).

When used alone, betanin was not cytotoxic to the HaCaT human keratinocyte cell line at varying concentrations; however, it substantially increased HaCaT cell survival following UVB exposure for various durations (Zand et al. 2024). Vitexin‐2‐O‐xyloside (XVX) has been identified as a powerful anti‐proliferative and pro‐apoptotic compound with potent cytotoxic activity against a range of human cancer cell lines (Papi et al. 2013). Betacyanins (BC), when combined with XVX, demonstrated synergistic upregulation of cytotoxicity after 24 h, without affecting normal keratinocyte NCTC 2544 cells. BC also showed a Schulte‐dependent anti‐proliferative effect in T24 bladder cancer cells (Scarpa et al. 2016). Different derivatives, such as betaxanthin (BX) and betacyanin (BC), exhibit hormetic cytotoxicity toward Caco‐2 cells and are highly effective at low doses (Farabegoli et al. 2017). Tryptophan‐betaxanthin most effectively decreased tumor size (56.4%), followed by betanin, phenylethylamine‐betaxanthin, and indicaxanthin (~26%–28%), whereas dopaxanthin and phenylalanine‐betaxanthin showed no effect (Henarejos‐Escudero et al. 2020). Similarly, L‐tryptophan ester‐betaxanthins suppress tumor growth by up to 43%, with efficacy comparable to that of cisplatin, whereas other derivatives achieve 31%–40% reductions (Henarejos‐Escudero et al. 2023).

### 3.5 Initiation of Apoptosis and Mitochondrial Dysfunction

Apoptosis, defined as programmed cell death, is a crucial component of anticancer activity. Betalains have been shown to influence apoptosis strongly, and most included studies have provided remarkable insights into their chemopreventive potential by promoting apoptosis in tumor cells. Betacyanins (BC) have shown potent pro‐apoptotic effects on T24 bladder cancer cells. BC strongly activates Caspase‐3 and Caspase‐8, indicating activation of the intrinsic apoptotic pathway in cancer cells, leading to apoptosis, mitochondrial dysfunction, and reduced ROS levels in normal cells (Saber et al. 2023). Another study on betanin demonstrated that inflammatory markers (COX‐2, TNF‐α, IL‐6, and NF‐κB) were reduced, and intracellular ROS levels increased in oral cancer cells (SCC131 and SCC4) (Zou et al. 2022). Betanin also protects against DNA damage under oxidative stress by inhibiting peroxynitrite‐dependent pathways and preventing H_2_O_2_‐induced DNA damage, which aligns with its antioxidant activity (Zand et al. 2024). In MG‐63 cells, betanin treatment results in elevated free radical production (ROS generation), as evidenced by the higher green fluorescence intensity (Liu et al. 2023). Betalains, in the form of betanin‐ZnO nanoparticles (NPs), reduced cellular nitric oxide (NO), elevated lipid peroxidation (TBARS), and decreased glutathione (GSH) concentration. They also substantially increased SOD and CAT enzyme activities, resulting in enhanced protection of cells against ROS‐induced damage (Hirad et al. 2025). Similarly, betaxanthin (BX) and betacyanin (BC) exhibited strong intracellular antioxidant activity in Caco‐2 cells, markedly inhibiting H_2_O_2_‐induced ROS production. Both BX and BC partially reduced the mRNA levels of the pro‐inflammatory markers COX‐2 and IL‐8, with BC exerting a stronger inhibitory effect on COX‐2 (Farabegoli et al. 2017). In ovarian cancer PA‐1 cells, (Shafreen and Kumar 2022) analyzed and reported elevated intracellular ROS generation in cells through bright 2′,7′‐dichlorofluorescein (DCF) fluorescence (Shafreen and Kumar 2022). Mechanistic investigations demonstrated that betalains might be responsible for causing apoptosis through mitochondrial pathways in cancer cells. Betalain treatment in glioma models increased reactive oxygen species (ROS) generation, mitochondrial membrane potential loss, mitochondrial swelling, and altered cytochrome c regulation, leading to caspase‐3 activation and apoptotic cell death (Kumorkiewicz‐Jamro et al. 2025). In another study, betalain dose‐dependently elevated apoptotic cell populations, with lung histopathology showing prominent apoptotic features and inflammatory infiltration in tumor tissues (Yin et al. 2021).

### 3.6 Modulation of Apoptotic and Cancer‐Related Gene Expression

Several studies (7 out of 10) have identified the role of betalains and their derivatives in modulating the expression of multiple cancer‐related genes at both the transcriptional and protein levels, particularly by upregulating pro‐apoptotic genes such as Bax and downregulating anti‐apoptotic genes such as BIRC5 and CTNNB1. In T24 bladder cancer cells, betaxanthin, a betalain derivative, significantly increased Bax expression and reduced BIRC5 (survivin) expression, thereby activating the intrinsic apoptotic pathway. In particular, betacyanin downregulates CTNNB1 (β‐catenin) expression, a gene associated with cell proliferation and survival (Scarpa et al. 2016). Betanin affects Bcl‐2 mRNA negatively while increasing Bax and caspase‐3/9 mRNA in the oral squamous cell carcinoma (OSCC) cell lines SCC131 and SCC4. It also mitigated PI3K and p‐Akt protein expression (Zou et al. 2022). Saber et al. demonstrated that betanin promotes pro‐apoptotic genes, including BAD (Bcl‐2‐associated death promoter (BAD)), Fas‐R, Caspase‐3, Caspase‐8, and Caspase‐9, and remarkably decreases anti‐apoptotic Bcl‐2 in HT‐29 and Caco‐2 cells.

**FIGURE 7:**
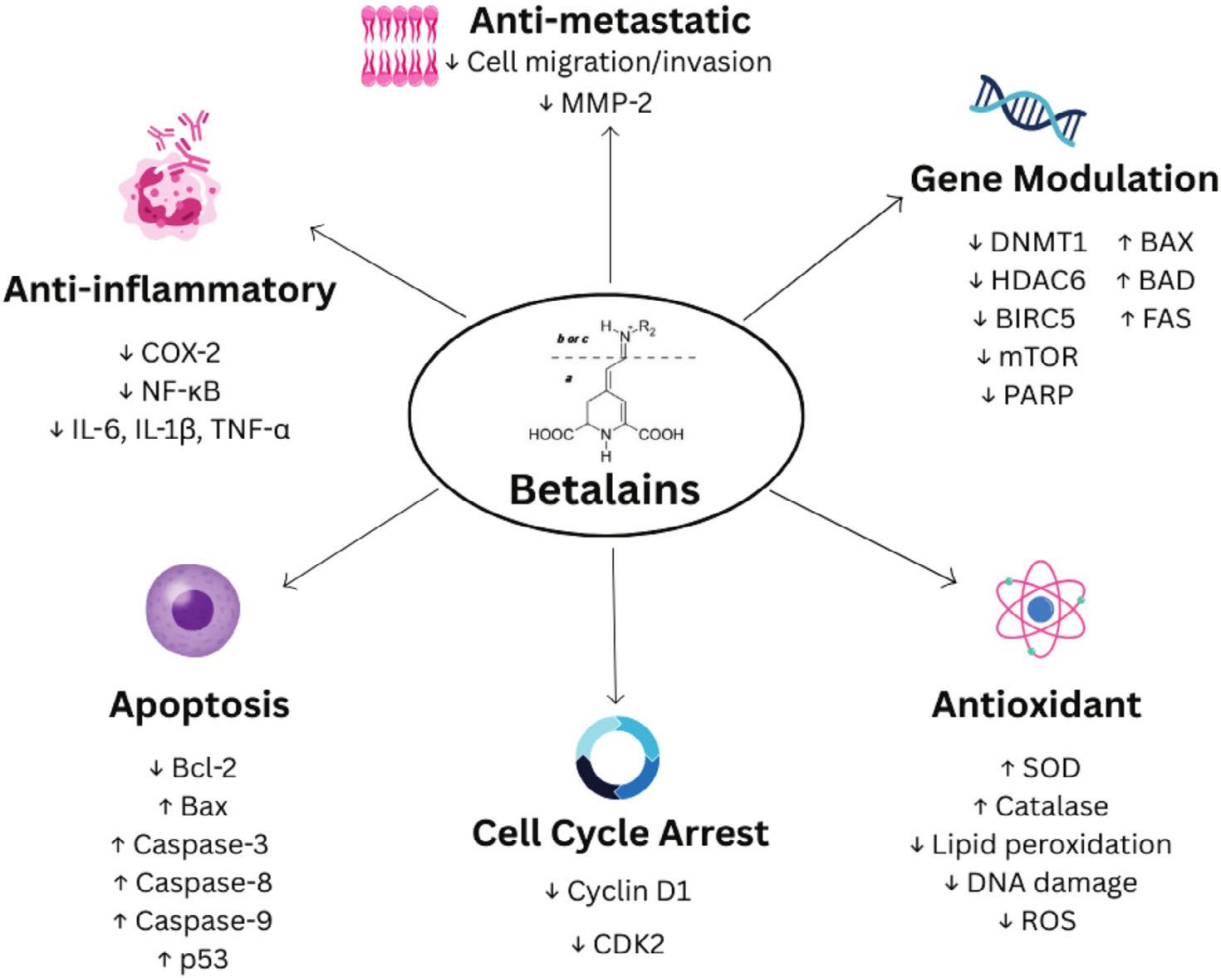
Molecular targets and gene expression regulated by betalains. This schematic diagram illustrates the diverse molecular mechanisms through which betalains exert biological effects, including anti‐inflammatory, antioxidant, pro‐apoptotic, and anti‐metastatic activities. It highlights the modulation of key signaling pathways and gene expression targets (e.g., BAX, Caspases) that collectively regulate cell survival, oxidative stress, and cell cycle progression. Downward arrows (↓): Inhibition or decreased expression; Upward arrows (↑): Indicate activation or increased expression.

Studies on different derivatives have shown that betaxanthin reduces Bcl‐2 levels, whereas betacyanin upregulates Bax levels (Farabegoli et al. 2017). Betanin, another derivative, inhibits the PI3K/AKT/mTOR signaling pathway, hindering cancer cell development by reducing the expression of p‐PI3K, PI3K, AKT, p‐AKT, mTOR, p‐mTOR, S6, and p‐S6 proteins in MG‐63 cells (osteosarcoma) (Liu et al. 2023). In lung cancer cells (A549), betanin‐ZnO NPs were used to enhance the compound's bioavailability and stability, resulting in increased apoptosis by reducing the anti‐apoptotic gene Bcl‐2 and promoting the pro‐apoptotic genes Bax and p53 (Hirad et al. 2025). Chemoprotective activity has been linked to the regulation of the insulin signaling pathway and the activity of the DAF‐16 transcription factor (Henarejos‐Escudero et al. 2023). Microarray analysis revealed the suppression of mTOR pathway genes (rict‐1, daf‐15) and over 2000 tumor‐related genes, resulting in the blockage of cell growth and proliferation signaling (Duraisamy et al. 2025). Betalains inhibit p‐PI3K, p‐Akt, mTOR, and cyclin D1, while elevating p53 and p21, reducing pro‐inflammatory cytokines (TNF‐α, IL‐6, IL‐1β), and regulating xenobiotic marker enzymes toward normal levels (Yin et al. 2021; Rusak et al. 2024). Zand et al. (Zand et al. 2024) conducted a study on skin cancer, in which betanin was found to decrease UVB‐induced upregulation of DNMT1, DNMT3A, and DNMT3B genes, which are involved in regulating gene expression (Zand et al. 2024). It also attenuates HDAC6 activation, which modulates cell viability. Betalains inhibit cell viability and proliferation by downregulating several key proliferative markers, including PI3K, AKT, and mTOR. Similarly, betalains induce apoptosis in cells by modulating apoptotic markers, including Bax, BIRC5, and p53. It also increases ROS production and modulates the expression of cancer‐related genes. Figure 7 helps visualize these interconnected effects and demonstrates the multitargeted anticancer potential of betalains.

### 3.7 Effects of Betalains on Inflammation

Betalains, particularly betanin, have demonstrated potent anti‐inflammatory effects across multiple experimental models by regulating key molecular pathways. The results demonstrate that betanin consistently suppresses pro‐inflammatory mediators, including TNF‐α, IL‐6, IL‐1β, IL‐8, iNOS, and COX‐2, initially by inhibiting NF‐κB signaling, while also elevating anti‐inflammatory cytokines, such as IL‐10 (Fernando et al. 2023; Wang et al. 2022). In colitis models, tomato‐derived betanin extracts have been shown to enhance body weight, decrease disease activity, and ameliorate colonic damage. However, potato‐derived extracts showed limited benefits, suggesting dose‐ and source‐dependent efficacy (Saito et al. 2023). In CPF‐induced liver injury, red beetroot extracts downregulate TNF‐α and IL‐1β at both the mRNA and protein levels, thereby regulating cytokine balance and demonstrating hepatoprotective effects (Albasher et al. 2019). Similarly, in paraquat‐induced nephrotoxicity, betanin supplementation downregulated NF‐κB, iNOS, and COX‐2, reduced nitrosative stress, and showed reno‐protective potential (Tan et al. 2015). In cardiac injury models, betanin administration suppressed NF‐κB and iNOS expression. It reduced MPO activity and LDL levels, thereby contributing to cardiopreventive effects alongside reductions in infarct size and oxidative stress (Yang et al. 2016). Furthermore, in intestinal epithelial cell studies, betanin and indicaxanthin have been shown to preserve barrier integrity by suppressing the release of IL‐6, IL‐8, and NO while activating the Nrf2/HO‐1 antioxidant signaling pathway, highlighting their potential as nutraceuticals (Wang et al. 2022; Smeriglio et al. 2021). Comparative findings further confirmed that betanin is the most effective betalain, with betaxanthins (vulgaxanthin I and indicaxanthin) exerting complementary roles through superior absorption and downregulation of free radical formation (Fernando et al. 2023; Wang et al. 2022). Collectively, these findings demonstrate that betanin is a promising bioactive compound with broad anti‐inflammatory and organ‐protective properties.

## 4 Discussion

### 4.1 Betalains' Stability and Their Pharmacokinetics

It is essential to review and understand the factors affecting betalain stability and pharmacokinetics, as this supports a clearer understanding of the paper. Nevertheless, it is important to clearly differentiate the distinct biological activities among betalain derivatives (Kumorkiewicz‐Jamro et al. 2021). For instance, betanin, a major betacyanin, is among the most studied and exhibits the strongest antioxidant potential and pro‐apoptotic effects in multiple cancer lines. In contrast, indicaxanthin, a betaxanthin, shows more significant anti‐inflammatory and redox‐modulating properties, particularly in noncancer and intestinal cell models. These activities result from structural differences between betacyanins and betaxanthins, which influence their cellular uptake, stability, and interactions with molecular targets, as previously discussed (Esquivel 2024). Betalain is a water‐soluble phytochemical found in 17 plant families. It offers health benefits, including antimicrobial and anticancer activity, as well as anti‐lipidemic, hepatoprotective, neuroprotective, cardiovascular, and anti‐inflammatory effects. Betalain also helps regulate intestinal motility and other outcomes. However, betalains are highly sensitive and unstable (Sadowska‐Bartosz and Bartosz 2021). They degrade through several mechanisms influenced by both intrinsic and extrinsic factors, which makes long‐term handling and storage difficult. The main factors responsible for betalain degradation are temperature, oxygen, and pH. Some metal cations, such as Fe2+, Fe3+, Cu2+, Al3+, Sn2+, and Cr3+, also reduce betalain stability and speed up degradation (Jurić et al. 2022). During degradation, neobetanins are formed. The pigment color changes from red or violet to yellow. This not only diminishes betalains' potential therapeutic properties but also restricts their use as food colorants (Wu et al. 2022). These influences shorten betalains' shelf‐life. For example, at pH 7 and 4°C, betalains are stable for 20 days. At −30°C, their shelf life extends to 275 days in frozen form (Vieira Teixeira da Silva et al. 2019). High temperature and oxygen exposure make betalains more acidic. Their pH can drop to 4.0–5.0, making them unsuitable for human use (Phuhongsung et al. 2020). Degradation changes the color of betalain, which is a disadvantage for use as a food coloring. It also reduces antioxidant properties by altering the phenolic ring and causing structural breakdown into betalamic acid and cyclo‐DOPA derivatives. These changes decrease free radical scavenging and antioxidant potential, and they reduce health benefits such as anti‐inflammatory, anticancer, hepatoprotective, and cardioprotective effects that depend on the core molecular structure (Naseer et al. 2019). In addition to stability, betalains have low bioavailability, which limits their physiological potential. Research shows that betanin is metabolized after passing through the pharynx and undergoing oxidative reactions in the oral cavity: 74% in the stomach wall, 60% in the colon, and 35% in the small intestine (Akbar Hussain et al. 2018). Betalains have unique pharmacokinetic properties due to their nitrogen‐containing structure and water‐soluble nature. After dietary intake, betalains such as betanin are partially absorbed in the small intestine. This shows that they enter systemic circulation in their intact form (Rahimi et al. 2019; Vieira Teixeira da Silva et al. 2019). Once they enter the bloodstream, betalains are distributed to organs such as the liver and kidneys. They bind free radicals, help reduce oxidative stress and inflammation, and assist in removing toxins from the body (Vieira Teixeira da Silva et al. 2019). Furthermore, a study was conducted on healthy humans (*n* = 8) to investigate the distribution of betanin and indicaxanthin in red blood cells (RBCs) from cactus pear fruit. The results showed that a much lower concentration of betanin (0.03 ± 0.005 μM) was detected in RBCs three hours after ingestion, while indicaxanthin peaked in RBCs at 1.03 ± 0.2 μM at three hours. Moreover, indicaxanthin remained in RBCs longer than betanin. Yet both pigments increased RBC resistance to oxidative damage and were undetectable after 12 h (Tesoriere et al. 2005). According to a human study, only 0.5%–0.9% of betalains were excreted via urine after consumption of red beet juices (Wiczkowski et al. 2018). Another study found that the food matrix did not affect indicaxanthin's absorption. However, betanin's absorption in beetroot was lower than in cactus pear (Tesoriere et al. 2013). This analysis shows that the source of betalains and the food matrix strongly affect betalain absorption and excretion. Hence, the bioavailability of betanin from various dietary sources, as measured by urinary excretion, is analyzed. Betanin from the cactus pear (*Opuntia* spp.) fruit showed a considerably greater bioavailability, estimated at 3.7% (Tesoriere et al. 2004). In comparison, red beet showed bioavailability of about 2.7%, 0.7%, or 0.28% (Esatbeyoglu et al. 2014; Allegra et al. 2014). These results highlight that different food matrices affect betalain bioaccessibility.

### 4.2 Betanin as the Multi‐Target Anticancer Phytochemical

Betanin exerts multifaceted anticancer activity by targeting multiple signaling pathways and cellular mechanisms, highlighting its value as a phytochemical for cancer prevention and treatment. The selected studies that met the inclusion criteria collectively demonstrated betanin, a betalain derivative and pigmented phytochemical derived from beetroot. Studies have demonstrated broad‐spectrum anticancer effects in human cancer cell lines, including colorectal (Saber et al. 2023; Farabegoli et al. 2017; Scarpa et al. 2016), skin (Zand et al. 2024), osteosarcoma (Liu et al. 2023), ovarian (Shafreen and Kumar 2022), oral (Zou et al. 2022), and lung (Chandrasekaran et al. 2022; Hirad et al. 2025) cancers. Betanin has shown potential benefits, mainly attributed to mechanisms such as cell cycle arrest (causing cells to stop proliferating), mitochondrial disruption (damaging mitochondria or preventing them from functioning), and induction of apoptosis (activating programmed cell death, also known as cell suicide). A study conducted by (Farabegoli et al. 2017) revealed that a combination of owvitexin‐2‐O‐xyloside (XVX) from *Beta vulgaris var. cicla* L. seeds, and betalains (R1 and R2) from *Beta vulgaris* var. rubra L. roots had significantly enhanced cytotoxicity, achieving 67.7% and 68.9% cell mortality, respectively, after 72 h against Caco‐2 colon cancer cells. This study suggests that betanin may significantly enhance the activity of other dietary flavonoids through mitochondrial dysfunction and oxidative stress‐mediated pathways. Similarly, another study investigated the anticancer effects of betanin and red beetroot hydroethanolic extracts (BHE) in Caco‐2 and HT‐29 colon cancer cell lines. The results showed that, in a dose‐dependent manner, IC50 values were 64 and 90 μg/mL for betanin and 92 and 107 μg/mL for BHE extracts, which induced apoptosis and reduced proliferation rates. The results were evidenced by DAPI (4′6‐Diamidino‐2‐phenylindole) staining and flow cytometry, which interprets the increase in pro‐apoptotic gene expression (gene‐encoded proteins that promote apoptosis) such as BAD, Caspase‐3, Caspase‐8, Caspase‐9, Fas‐R, and also a decline in the level of B‐cell lymphoma 2 (Bcl‐2; a protein that plays an important role in preventing apoptosis) (Saber et al. 2023). Along with the initiation of apoptosis, the modulation of key apoptotic proteins such as Bax, Bcl‐2, and Caspase‐9, as well as betanin, also exhibits anti‐proliferative effects. This effect is mediated by other mechanisms, including reactive oxygen species (ROS) generation (Liu et al. 2023; Kumorkiewicz‐Jamro et al. 2025), modulation of mitochondrial membrane potential (Zou et al. 2022; Shafreen and Kumar 2022), and DNA fragmentation (Zou et al. 2022; Saber et al. 2023; Chandrasekaran et al. 2022; Hirad et al. 2025; Liu et al. 2023; Scarpa et al. 2016; Zand et al. 2024; Kumorkiewicz‐Jamro et al. 2025). Betanin also regulates oncogenic signaling pathways, such as NF‐κB/PI3K/Akt, PI3K/Akt/mTOR/S6, and Wnt/β‐catenin, to achieve desirable outcomes. Both in vitro and in vivo studies have demonstrated that betalains have promising anti‐cancer potential. The study shows that in *Caenorhabditis elegans* models, downregulation of tumor development was observed by regulating gene expression associated with cancer and by maintaining an oxidative balance involving tryptophan‐betaxanthin and related betalains (Henarejos‐Escudero et al. 2023, 2020). In animal‐based cancer models, betanin has demonstrated chemoprotective effects against oral squamous cell carcinoma, with both in silico and experimental analyses indicating that it downregulates tumor progression (Duraisamy et al. 2025). Overall, these findings demonstrated that betalains are natural compounds that play a significant role in targeting anticancer mechanisms.

### 4.3 Modulation of Oncogenic Signaling Pathways

**FIGURE 8:**
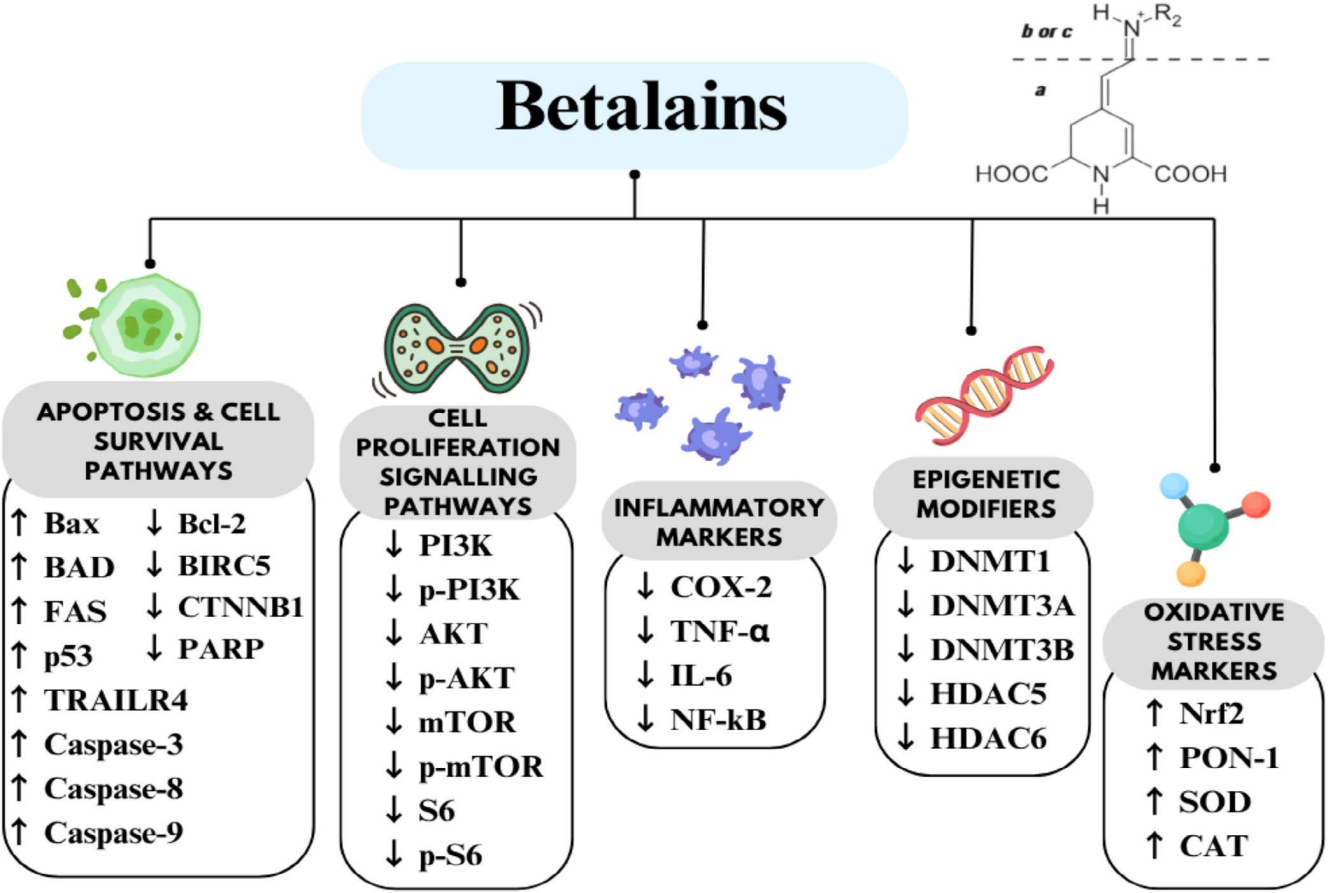
Schematic representation of modulation of oncogenic signaling pathways. Downward arrows (↓): Inhibition or decreased expression; upward arrows (↑): Indicate activation or increased expression.

Several research papers have observed and discussed, as a recurring finding, that betanin significantly impacts critical signaling pathways involved in cancer progression (Liu et al. 2023; Kumorkiewicz‐Jamro et al. 2025). Furthermore, (Shafreen and Kumar 2022) reported decreased proliferation markers and increased apoptosis levels in PA‐1 cells from osteosarcoma and ovarian cancer (Shafreen and Kumar 2022; Liu et al. 2023; Kumorkiewicz‐Jamro et al. 2025). The study reported that betanin inhibits the PI3K/AKT/mTOR/S6 pathway in MG‐63 osteosarcoma cells, leading to reduced cell proliferation and induction of apoptosis (Liu et al. 2023; Kumorkiewicz‐Jamro et al. 2025). In addition, a 2021 study found that betalain treatment inhibits the proliferation of nonsmall cell lung cancer cells by targeting the PI3K/Akt/mTOR pathway, a critical signaling cascade implicated in tumor growth and cell proliferation (Yin et al. 2021). In OSCC, betanin exerts an anti‐inflammatory effect by reducing cytokine levels and suppressing the NF‐κB/PI3K/Akt signaling pathway (Zou et al. 2022). A similar response was observed in an in vitro model, as betanins block the NF‐κB pathway while activating the COX pathway in fibroblasts and immune cells (Rusak et al. 2024). Studies in both colorectal and lung cancer models have provided evidence that betanin upregulates the expression of pro‐apoptotic genes (e.g., Bax, Caspase‐3) and reduces levels of anti‐apoptotic proteins such as Bcl‐2 (Saber et al. 2023; Chandrasekaran et al. 2022; Hirad et al. 2025; Scarpa et al. 2016). Additionally, the combination of betanin with vitexin‐2‐O‐xyloside (XVX) in bladder and colon cancer cells has been shown to increase pro‐apoptotic signaling and reduce survival markers, including BIRC5 (survivin) and CTNNB1 (β‐catenin) (Farabegoli et al. 2017; Scarpa et al. 2016). A study (Farabegoli et al. 2017) reported that a triple combination of XVX, R1, and R2 provided the highest cytotoxicity against Caco‐2 colon cancer cells. As a result, it decreases COX‐2, IL‐8, and Bcl‐2 levels, enhances pro‐apoptotic Bax expression, and reduces anti‐apoptotic BIRC5 and CTNNB1. Betalains also interact with principal signaling pathways such as NF‐κB and PI3K/Akt/mTOR, which are involved in inflammation, cell proliferation, and cell death responses in cancer cells. It has been shown that betalains, especially betanin, can indirectly regulate these pathways by scavenging reactive oxygen species (ROS), which have been shown to stimulate NF‐kB and PI3K/Akt/mTOR signaling (Yin et al. 2021; Rusak et al. 2024). Betalains inhibit activation of these pro‐inflammatory and pro‐survival pathways by reducing ROS levels, slowing tumor progression, and inducing apoptosis. Moreover, betalains can directly suppress kinases in these pathways by decreasing the expression of PI3K, AKT, and mTOR proteins in osteosarcoma and oral cancer cell lines (Zou et al. 2022; Liu et al. 2023). This twofold mechanism: indirect via ROS scavenging, and direct via kinase inhibition, is an improvement of the chemopreventive potential of betalains. Figure 8 illustrates the role of betanin in modulating apoptosis, cell proliferation, inflammation, epigenetic modifications, and oxidative stress, all of which may contribute to its anticancer and protective properties.

### 4.4 Nanotechnology and Other Advanced Approaches Enhanced Efficacy

A revolutionary approach, Nanotechnology‐based carriers, was reviewed in selected studies, highlighting the potential benefits of betanin‐coated ZnO nanoparticles. This approach has been observed to improve the stability, bioavailability, and targeted delivery of phytochemicals, including betalains. Among these systems, zinc oxide nanoparticles (ZnO‐NPs) are the centre of attention due to their biocompatibility, large surface area, and their ability to elevate cellular uptake of bioactive compounds. A survey by Hirad et al. (2025) reported that this nano‐formulation yields promising results against A549 lung carcinoma cells, exhibiting enhanced cytotoxic and antioxidant activity compared to free betanin. Moreover, it highlights the importance of focusing on nanotechnology as it has improved the delivery, bioactivity, and stability of betanin in cancer treatment (Hirad et al. 2025). Overall, this approach would yield the most promising results. Yet, it is not the only viable strategy, as it is neither cost‐effective nor scalable for enhancing betalain delivery in clinical or nutraceutical applications. Therefore, other approaches, such as encapsulation and polymer‐based carriers, are considered significant, as they improve the stability and bioavailability of betalains without the potential toxicity concerns associated with some nanomaterials (Koop et al. 2022). Encapsulation is one of the most widely studied strategies for maintaining betalain stability, as it protects against degradation caused by factors such as light, oxygen, temperature, and pH fluctuations. Not only that, but this system also protects betalains during gastrointestinal digestion, leading to improved controlled release and increased bioavailability (Calva‐Estrada et al. 2022). Another cost‐effective and clinically viable approach is complex formation, as a study reported that antioxidant metals, such as selenium, increase the bioavailability of betalains, thereby stabilizing them. Ascorbic acid primarily facilitates the formation of the betalain‐Se complex. These complexes are absorbed via a Se absorption route. However, it is preferable to have organic Se rather than free inorganic Se to achieve a significant increase in bioavailability (Khan and Giridhar 2015). Hence, proven nanotechnology may enhance the pharmacological potential of betalains. Still, encapsulation and other formulation technologies are more reliable, as they are cost‐effective and scalable alternatives to improve betalain delivery in clinical or nutraceutical applications.

### 4.5 Protection Against DNA Damage

Studies have shown that betalains possess chemopreventive properties that protect healthy or treated cells from DNA damage while inducing cytotoxicity in cancer cells. Betanin reduces UV‐induced DNA fragmentation in HaCaT keratinocytes (skin cells). Before discussing the study, it's important to know that HaCaT cells are an immortalized human keratinocyte cell line. These cell lines are considered nontumorigenic models commonly used to represent normal skin cells rather than skin cancer cells; therefore, the researcher has presented them as evidence of cytoprotective and chemopreventive effects rather than direct anticancer activity. Additionally, it regulates epigenetic regulators such as DNA Methyltransferases (DNMTs) and histone deacetylases (HDACs), which have been implicated in skin cancer prevention. It can regulate the expression of antioxidant enzymes, filter free radicals, and downregulate oxidative stress caused by xenobiotics (Zand et al. 2024). Betanin inhibits DNA methyltransferase and induces DNA damage in human cancer cells. (Liu et al. 2023; Martinez et al. 2020). Betanin also induces apoptosis in cancer cell lines, as reported in previous studies on osteosarcoma, leukemia, and colon cancer cells. It also downregulates signaling pathways such as PI3K/AKT/mTOR/S6, which are responsible for cell growth and proliferation (Liu et al. 2023).

### 4.6 Synergistic Effects With Other Phytochemicals

These studies suggest that betanin has chemoprotective potential that can be enhanced when combined with other phytochemicals. (Farabegoli et al. 2017) Evaluated the effect of combining betacyanins (BC) sourced from beetroot and vitexin‐2‐O‐xyloside (XVX) on T24 bladder cancer cell growth, and found that this combination suppressed cell growth more effectively than BC alone. This combination has been shown to increase pro‐apoptotic Bax expression, reduce cancer cell survival markers such as BIRC5 (survivin) and CTNNB1 (β‐catenin), and regulate Caspase‐8 activity, thereby inducing extrinsic apoptosis (Scarpa et al. 2016). Similarly, against the colorectal cancer cells (Caco‐2), when dosed with the combination of XVX, BC, and BX, results in elevating collaborative cytotoxic effects and upheld over 72 h, suppressing COX‐2 and IL‐8 expression, significantly regulating multidrug resistance (MDR) mechanisms, and decreasing Bcl‐2 levels; such a response was not recorded with XVX alone (Farabegoli et al. 2017). These studies underscore the importance of exploring novel phytochemical combinations with betanin to enhance its efficacy in cancer therapy.

### 4.7 Betalains as Anti‐Inflammatory Effects

Mammalian studies have consistently demonstrated the anti‐inflammatory properties of betalains in various organs. Betanin supplementation may reduce inflammatory cytokine levels and oxidative stress markers in paraquat‐treated rat kidneys, thereby demonstrating its significant nephroprotective potential. (Tan et al. 2015). Similarly, betanin enhanced myocardial infarction in rats by modulating inducible nitric oxide synthase (iNOS), myeloperoxidase activity, and lipid peroxidation (Rusak et al. 2024; Martinez et al. 2020). Hence, these results highlight betanin's cardioprotective capacity by downregulating oxidative and inflammatory responses. (Yang et al. 2016; Martinez et al. 2020). The hepatoprotective role of *Beta vulgaris* root extract is supported by evidence, as it regulates chlorpyrifos‐induced oxidative stress and recovers liver injury by suppressing pro‐inflammatory mediators (Albasher et al. 2019). The inflammatory process is an important component of the tumor microenvironment that facilitates cancer development by promoting cell growth, survival, and metastasis. Chronic inflammation may also create an environment conducive to tumor development and resistance to treatment. Betalains (anti‐inflammatory properties) may also be used to regulate this inflammatory response by decreasing levels of pro‐inflammatory cytokines and by inhibiting pathways such as NF‐kB and COX‐2. Betalains can alter the tumor microenvironment by modulating inflammation, likely slowing cancer progression and enhancing the effectiveness of cancer treatments.

### 4.8 Betalains and Inflammation Pathways

Regardless of whether the study was performed in vivo or in vitro, betalains demonstrated significant anti‐inflammatory effects. At the cellular level, two betalain derivatives, betacyanins and betaxanthins, have shown that structural differences among betalains influence their immunomodulatory effects by suppressing the production of IL‐6, IL‐1β, iNOS, and COX‐2 via NF‐κB inhibition. Betanin was the most effective in boosting IL‐10, whereas betaxanthins showed weaker effects (Fernando et al. 2023). Similarly, intestinal Caco‐2 cells efficiently absorb betanin, vulgaxanthin I, and indicaxanthin, demonstrating immunomodulatory effects by reducing the pro‐inflammatory cytokines IL‐6 and IL‐8, indicating gut‐associated immune regulation (Wang et al. 2022). Additionally, a study demonstrated that intestinal inflammation was reduced more effectively by prickly pear betalain‐rich extracts than by isolated betalain compounds. They downregulate IL‐6, IL‐8, and NO, recover epithelial barrier integrity, and block NF‐κB signaling while also presenting good stability, bioaccessibility, and intestinal absorption potential (Smeriglio et al. 2021). Studies show that betalains have a significant effect on macrophage polarization, a crucial process that regulates inflammation in the tumor microenvironment. Betalains have been reported to suppress pro‐inflammatory mediators, including COX‐2 and iNOS, which are generally associated with M1 macrophage polarization, a phenotype that promotes inflammation and tumor progression (Fernando et al. 2023; Wang et al. 2022; Salimi et al. 2021). By regulating these markers, betalains can potentially shift macrophage polarization toward the M2 phenotype, which is involved in tissue repair and anti‐inflammatory responses (Ruivo et al. 2024). Such a transition can aid in minimizing inflammation sustained over time, which enables the development of cancer, and thus possibly enhances the overall anti‐inflammatory environment and diminishes the aversive consequences of inflammation in the development of cancer.

Betanin supplementation has the potential to decrease inflammatory cytokine levels and mitigate oxidative stress markers in paraquat‐treated rat kidneys, thereby demonstrating the significant nephroprotective potential of betanin (Tan et al. 2015). Similarly, betanin enhanced myocardial infarction in isoproterenol‐treated rats by modulating inducible nitric oxide synthase (iNOS), myeloperoxidase activity, lipid peroxidation, and LDL oxidation, thereby reducing tissue damage. Hence, these results highlight the cardioprotective capacity of betanin through the downregulation of oxidative and inflammatory responses (Yang et al. 2016). The hepatoprotective role of *Beta vulgaris* root extract is supported by evidence that it regulates chlorpyrifos‐induced hepatotoxicity and oxidative stress, and recovers liver injury by suppressing pro‐inflammatory mediators (Albasher et al. 2019). Collectively, the findings of these selected studies demonstrated that betalains exhibit protective and preventive actions through multiple inflammation‐related pathways, affecting the cardiovascular, renal, hepatic, intestinal, and immune systems.

### 4.9 Betalains Action in Cancer and Inflammation: A Critical Discussion of Its Mechanisms of Action

Among the betalains (Betanin), especially those derived from beetroot, considerable interest has been shown due to their potential for treating cancer and inflammation. Although much research focuses on the direct impact of betalains, appreciation of the complex processes of action of these compounds is increasing. The vast majority of the research used high concentrations of betalains, and they usually did not differentiate their contributions to whether the action was caused by betalains or by metabolites, redox regulation, or alterations in the extracellular microenvironment (Smeriglio et al. 2021).

#### 4.9.1 Direct and Indirect Effects of Beetroot Betalains

Betalains, especially betanin, are reportedly common in investigations of the anticancer effects of beetroot extract on several cell lines. In these studies, high concentrations of betanin are frequently used and have been shown to reduce oxidative stress, inhibit cell growth and trigger apoptosis. For example, A549 lung cancer cells treated with betanin exhibit reduced cell viability, increased apoptosis, and gene expression changes associated with apoptosis (e.g., Bax and P53) (Tan et al. 2015). On the same note, betanin inhibits cell growth and alters mitochondrial membrane potential in ovarian cancer models (Albasher et al. 2019). These effects, however, cannot be attributed solely to direct interactions with cancer cells. One should note that betanin is converted in vivo, and such products may play a role in its biological effect. Moreover, it is well established that betanin has antioxidant properties, which can be instrumental in counteracting the oxidative stress associated with cancer development (Tan et al. 2015).

In addition to direct effects, betanin's anti‐inflammatory effects may be mediated indirectly through redox regulation. Betanin was found to affect cellular redox homeostasis, inducing the expression of antioxidant enzymes, such as heme oxygenase‐1 (HO‐1) (Fernando et al. 2023). This antioxidant‐controlling property, specifically its radical‐scavenging effect, suggests that betanin might alleviate the oxidative stress to which the tumor microenvironment is readily subject and, subsequently, prevent cancer cell proliferation (Smeriglio et al. 2021). Besides, the anti‐inflammatory properties of betanin have been associated with the inhibition of pro‐inflammatory cytokines, such as cyclooxygenase‐2 (COX‐2) and inducible nitric oxide synthase (iNOS), which promote inflammation and tumor cell development (Yang et al. 2016). This suggests that the anticancer action of betanin, as observed, might be partially mediated by its capacity to regulate the tumor's inflammatory status.

#### 4.9.2 Other Sources of Betalains: Mechanisms of Action Reminiscent

Other plant species, including prickly pear and amaranth, also contain betalains, and it has been shown that betalains of the latter can have an identical anticancer and anti‐inflammatory effect as those of plant sources. Indicaxanthin and vulgaxanthin I are prickly pear betalains, which inhibit the production of reactive oxygen species (ROS) and pro‐inflammatory cytokines such as IL‐6 and IL‐8 in an in vitro model of intestinal inflammation (Smeriglio et al. 2021). These betalains exhibit strong antioxidant and anti‐inflammatory activity and can be considered for the treatment of diabetic bowel disease due to their significant potential (Smeriglio et al. 2021). Interestingly, although betacyanins (betanin) and betaxanthins (indicaxanthin) exhibit anti‐inflammatory effects, their mechanisms differ significantly. Betacyanins, including betanin, were also found to be antioxidants, directly reducing ROS production and regulating redox signaling through other pathways (Henarejos‐Escudero et al. 2023; Salimi et al. 2021; Martinez et al. 2020). Alternatively, betaxanthins such as indicaxanthin exhibit anti‐inflammatory effects but, at higher doses, pro‐oxidant effects, contributing to ROS production in macrophage models (Fernando et al. 2023). This emphasizes the concentration and the possibility of varying biological responses across the particular betalain subclass.

#### 4.9.3 Indirect Effects: Sensitizing the Extracellular Microenvironment

The impact of betalains on the extracellular microenvironment is also essential to their anticancer and anti‐inflammatory actions. It has been demonstrated that betalains can affect cytokine production, immune cell function, and the overall inflammatory response. In oral cancer models, e.g., betanin can modulate the NF‐kB pathway, which is central to regulating inflammation and the immune response (Duraisamy et al. 2025). In the same vein, betalains have been shown to reduce oxidative stress and inflammatory responses in animal models of organ damage, including liver and kidney injury, by modulating intracellular and extracellular signaling (Tan et al. 2015; Albasher et al. 2019; Martinez et al. 2020). In addition, betanin has also been reported to act not only through direct cell interaction but also by regulating the tumor microenvironment, which comprises inflammatory cytokines, ROS, and extracellular matrix‐associated components (Yang et al. 2016). This indicates that betalains have direct and indirect therapeutic effects through their combined antioxidant, anti‐inflammatory, and redox‐regulating activities.

### 4.10 Strengths and Limitations of Review

The key strength of this systematic review is that it focuses only on controlled in vitro and in vivo studies, examines the cellular level, and provides specific evidence of the anticancer and anti‐inflammatory potential of betalains. This selection criterion facilitates a better understanding of the effects of betalains on underlying mechanisms, such as the initiation of apoptosis, the reduction of inflammation, the modulation of mitochondrial dysfunction and oxidative stress, and gene regulation, particularly in the context of cancer. Moreover, this review included studies from various geographical regions. It discusses multiple cell lines across different cancer types, including colorectal, lung, ovarian, breast, and bladder cancers, highlighting the broadening scope of findings. Adherence to the PRISMA guidelines further enhances the credibility and reliability of this review.

However, this study has some limitations that must be recognized. (1) The studies included all focused on in vitro and in vivo models, which lack the mechanism of action regarding human‐based trials. (2) Heterogeneity was observed in the experimental designs of the selected studies, including dose specification, time duration, purity, and specific cancer cell lines, which diversifies the comparison and quantitative analysis of results. (3) The lack of uniformity of the betanin source and purity causes potential variability in bioactivity results reported across studies. (4) One of the major limitations of this systematic review was the decision to restrict the selection of studies to a time frame between 2015 and 2025. However, this time frame was chosen to narrow the scope and, in particular, to ensure that the review incorporates the most current research; it may have excluded studies from the early 2010s that could contain valuable mechanistic insights to help explain the chemopreventive perspective on betalains. Some earlier studies may have provided foundational knowledge that influenced more recent work, particularly in elucidating the molecular and systematic mechanisms underlying betalains' anticancer and anti‐inflammatory activities. Hence, future research should expand the study criteria's time frame to include a broader range of studies, particularly those that have contributed to the initial characterization of betalain's bioactive properties. (5) The review also mentions a few references to other phytochemicals influencing the activity of betalains, limiting a better understanding of their role in combinatorial therapies, as they were briefly explored. (6) Lastly, the limitation of this review is that not all of the studies could be accessed because of paywalls, institutional access, or the articles were published in nonopen access journals. These studies were not accessible despite the vast efforts, such as employing alternative methods of access and reaching out to authors. Although most of these articles were retrieved through additional sources, the studies that were not retrieved might pose a potential bias, especially when they did not have similar results or research designs. This omission may have implications for the thoroughness of evidence and the strength of the conclusions made in the review. Overall, this review highlights key findings and valuable insights into betalains' anti‐cancer mechanisms, providing comprehensive in vitro and in vivo data. At the same time, clinical investigations are still necessary to confirm their chemopreventive benefits and safety in the human population.

### 4.11 Variability in Response Across Cell Lines

While most selected studies report strong anti‐proliferative and pro‐apoptotic effects of betalains, some have shown limited, indirect effects in specific cell lines. Studies using nontumorigenic models, such as keratinocyte cell lines, have primarily focused on the cytoprotective and epigenetic modulation of beetroot red pigment (betalains) rather than on direct anticancer activity (Zand et al. 2024). Similarly, some other studies included in this review used inflammatory or oxidative stress models to analyze the properties of betalains, yet no direct reflection of cytotoxic effect was reported against cancer cells, although they provided mechanistic relevance (Fernando et al. 2023; Wang et al. 2022; Smeriglio et al. 2021). Moreover, some studies reported inconsistencies in molecular responses, suggesting that the beetroot red pigment has different effects across cell types, experimental conditions, and dosages (Zou et al. 2022).

Hence, these findings concluded that betalain has a heterogeneous, context‐dependent nature, highlighting the need for cautious interpretation of its potential as an anticancer agent. Therefore, it is recommended to use standardized, clinically relevant models for further studies.

### 4.12 Closing Statement of Discussion

The collective key findings of these twenty‐one studies strongly associate betalains with their promising potential as safe, economical, and natural nitrogenous compounds with multifaceted anticancer properties, mediated through biological pathways such as mitochondrial pathways, oxidative stress induction, inflammation suppression, and epigenetic regulation. However, its efficacy can be enhanced through new techniques, such as nanotechnology, or by combining it with other bioactive compounds. It is important to note that most studies included in this systematic review were conducted in in vitro *cell* culture models, which provide only preclinical evidence rather than confirmed therapeutic effects in humans. Hence, a comprehensive human‐based clinical validation remains a gap in validating the therapeutic potential and clinical application of betanin.

### 4.13 Research Gaps and Future Directions

Preclinical studies, particularly in vitro and in vivo, have strongly demonstrated the chemoprotective efficacy of betalains; however, there is a gap in assessing their potential in animal models and clinical trials. Therefore, more focus is required on these core areas.

- Pharmacokinetic Parameter Determination: to adjust the dosage and to determine the metabolic fate;
- Human Clinical trials: Explore its interaction with conventional therapy, along with which the human body's response is generated;
- A comparison between the natural‐based dietary resource, betalain, and pharmacological supplementation still needs to be explored.

A vital limitation of these studies is the lack of uniform betalain content, and the extract's purity remains in a gray area, resulting in inconsistent findings. Furthermore, longitudinal multi‐omics analyses incorporating p53‐dependent mechanisms are needed to elucidate better how betanin influences cancer heterogeneity and molecular pathways.

A significant evaluation is needed to determine whether intact betalain and its derivatives have chemoprotective potential once the drug is developed. In addition, betalains associated with the Nrf2 pathway and Phase II detoxifying enzymes should be prioritized to leverage their antioxidant properties. Lastly, the response in the progression or suppression of any hormone still needs clarification, as current studies have shown dose‐dependent responses, and more phytochemical combinations need to be explored to develop nutraceuticals or functional foods enriched with betanin and other phytochemicals.

### 4.14 Betalains and Their Importance in Food Science

Most natural sources play a role in food science and serve as therapeutic agents as well (Butt et al. 2013). Betalains occur naturally and are found predominantly in plants such as beets ( *Beta vulgaris* ), cactus pear (*Opuntia* spp.), swiss chard, and amaranth. These plants are not only valued for their vibrant colors but also for their bioactive properties, including antioxidant, anti‐inflammatory, and anticancer effects. In food science, betalains are increasingly used as natural colorants and functional ingredients in health‐promoting foods (Choo 2019). Their bioavailability and stability during food processing are key factors in determining their effectiveness when consumed. Betalains are sensitive to processing conditions such as heat, light, and pH, which can affect their stability and potency (Kumorkiewicz‐Jamro et al. 2021). However, betalains derived from these natural sources continue to be explored for their potential in functional foods, offering a natural means to enhance the therapeutic and nutritional value of everyday foods.

## 5 Conclusion

This systematic review has analysis the included studies related to the potential of betalains as anticancer and anti‐inflammatory properties, including both in vitro and in vivo evidence. The results of the analysis suggest that betalains, especially betalain‐rich compounds, may show a positive exert biological effects like regulation of oxidative stress, induction of apoptosis in certain cancer cell models, and blocking of inflammatory mediators. Even though such promising results are observed across all the studies, these effects can not be generalized due to inconsistency and changes in effect because of substantial heterogeneity in compound type, extraction methods, experimental models, and dosing protocols. Moreover, a major proportion of the evidence is from in vitro studies with limited validation or from in vivo studies with insufficient clinical data. In addition, many included studies report an indirect potential of betalains as anti‐inflammatory activities rather than direct cytotoxic effects against cancer cells. Although some selected studies aim to enhance the stability and bioactivity of betalains by advance approaches, such as nanoparticle‐based delivery systems, these remain at early experimental stages. These limitations bind current evidence to be insufficient to support firm and standarize conclusions regarding the safety, efficacy, or clinical applicability of betalains as therapeutic agents. The standardized experimental designs, dose optimization, and well‐controlled in vivo and clinical studies should be focused on clarifying betalains' potential role against cancer in future research.

## Author Contributions

**Ahmad Mujtaba Noman:** validation, visualization, writing – review and editing. **Zoha Saleem:** investigation. **Nimra Anees:** data curation, software. **Hassan Raza:** writing – review and editing, supervision, resources. **Farhang Hameed Awlqadr:** resources, supervision, conceptualization, writing – review and editing, writing – original draft. **Muhammad Tauseef Sultan:** methodology, formal analysis, writing – review and editing. **Khaled Arab:** funding acquisition, project administration. **Aroob Fatima:** investigation, data curation, writing – original draft.

## Funding

The authors have nothing to report.

## Ethics Statement

The authors have nothing to report.

## Consent

The authors have nothing to report.

## Conflicts of Interest

The authors declare no conflicts of interest.

## Supporting information


**Appendix S1:** List of excluded studies that were retrieved (*n* = 76).
**Appendix S2:** Risk of Bias Assessment for in vivo studies using the SYRCLE Tool (*n* = 09).
**Appendix S3:** Domains related to the SYRCLE Tool.
**Table S1:** PRISMA checklist of included studies.
**Table S2:** Risk of bias assessment of in vitro studies using a customized tool (*n* = 12).
**Table S3:1** Risk of Bias Assessment of inflammation‐related in vitro studies using the QUIN Tool (*n* = 05).
**Table S3:2** Domains related to the QUIN Tool.

## Data Availability

All raw data are available upon request from the corresponding authors.
