# Supplementary material for: Anticancer and Anti‐Inflammatory Potential of Betalains: A Systematic Review on Preclinical Studies
Source: Food Sci Nutr. 2026 Jul 24;14(7):e72133. doi: 10.1002/fsn3.72133 (PMC13397872; doi:10.1002/fsn3.72133)
Supplement: Supplementary file 1 — Appendix S1: List of excluded studies that were retrieved (n = 76). Appendix S2: Risk of Bias Assessment for in vivo studies using the SYRCLE Tool (n = 09). Appendix S3: Domains related to the SYRCLE Tool. Table S1: PRISMA checklist of included studies. Table S2: Risk of bias assessment of in vitro studies using a customized tool (n = 12). Table S3:1 Risk of Bias Assessment of inflammation‐related in vitro studies using the QUIN Tool (n = 05). Table S3:2 Domains related to the QUIN Tool. [file FSN3-14-e72133-s001.docx]

**Supplementary Appendix S1:** List of Excluded Studies that were Retrieved (n = 76).

| **No.** | **Title of the Study** | **DOI** | **Reason for Exclusion** |
| --- | --- | --- | --- |
| **1** | Evaluation of the IKKβ Binding of Indicaxanthin by Induced-Fit Docking, Binding Pose Metadynamics, and Molecular Dynamics. | <https://doi.org/10.3389/fphar.2021.701568> | In silico study design present |
| **2** | Indicaxanthin Induces Autophagy in Intestinal Epithelial Cancer Cells by Epigenetic Mechanisms Involving DNA Methylation. | <https://doi.org/10.3390/nu15153495> | In silico study design present |
| **3** | Epigenetic Remodeling of Regulatory Regions by Indicaxanthin Suggests a Shift in Cell Identity Programs in Colorectal Cancer Cells. | <https://doi.org/10.3390/ijms26136072> | In silico study design present |
| **4** | Characterization of Extractable and Non-Extractable Phenols and Betalains in Berrycactus (Myrtillocactus geometrizans) and Its Chemoprotective Effect in Early Stage of Colon Cancer In Vivo. | <https://doi.org/10.3390/antiox13091112> | Wrong intervention |
| **5** | Red-Beet Betalain Pigments Inhibit Amyloid-β Aggregation and Toxicity in Amyloid-β Expressing Caenorhabditis elegans. | <https://doi.org/10.1007/s11130-022-00951-w> | Alzheimer disease |
| **6** | Anti-Amyloid β Aggregation Activity and Cell Viability Effect of Betacyanins from Red Pitahaya (Hylocereus polyrhizus) for Alzheimer's Disease. | <https://doi.org/10.1007/s11130-023-01081-7> | Alzheimer disease |
| **7** | Therapeutic Application of Betalains: A Review. | <https://doi.org/10.3390/plants9091219> | Review |
| **8** | Root Vegetables-Composition, Health Effects, and Contaminants. | <https://doi.org/10.3390/ijerph192315531> | Review |
| **9** | The potential role of dietary plant ingredients against mammary cancer: a comprehensive review. | <https://doi.org/10.1080/10408398.2020.1855413> | Review |
| **10** | A Narrative Review on the Potential of Red Beetroot as an Adjuvant Strategy to Counter Fatigue in Children with Cancer. | <https://doi.org/10.3390/nu11123003> | Review |
| **11** | Opuntia ficus-indica (L.) Mill. - anticancer properties and phytochemicals: current trends and future perspectives. | <https://doi.org/10.3389/fpls.2023.1236123> | Review |
| **12** | Beetroot as a novel ingredient for its versatile food applications. | <https://doi.org/10.1080/10408398.2022.2055529> | Review |
| **13** | Pink pressure: beetroot (Beta vulgaris rubra) as a possible novel medical therapy for chronic kidney disease. | <https://doi.org/10.1093/nutrit/nuab074> | Review |
| **14** | Biological Activities of Plant Pigments Betalains. | <https://doi.org/10.1080/10408398.2012.740103> | Review |
| **15** | Underutilized plants of the Cactaceae family: Nutritional aspects and technological applications. | <https://doi.org/10.1016/j.foodchem.2021.130196> | Review |
| **16** | Antioxidant Capacity, Antitumor Activity and Metabolomic Profile of a Beetroot Peel Flour. | <https://doi.org/10.3390/metabo13020277> | Review |
| **17** | Phytoconstituents and pharmaco-therapeutic benefits of pitaya: A wonder fruit. | <https://doi.org/10.1111/jfbc.13260> | Review |
| **18** | Extraction, stabilization, and health application of betalains: An update. | <https://doi.org/10.1016/j.foodchem.2025.144011> | Review |
| **19** | Beetroot Bioactive and its Associated Health Benefits: Considerations for Utilization of Beetroot in Value-added Products. | <https://doi.org/10.2174/2772574X14666230725110541> | Review |
| **20** | A comprehensive review of beetroot (Beta vulgaris L.) bioactive components in the food and pharmaceutical industries. | <https://doi.org/10.1080/10408398.2022.2108367> | Review |
| **21** | Betalain health-promoting effects after ingestion in Caenorhabditis elegans are mediated by DAF-16/FOXO and SKN-1/Nrf2 transcription factors. | <https://doi.org/10.1016/j.foodchem.2020.127228> | Irrelevant outcome |
| **22** | The synergistic effects of betanin and radiotherapy in a prostate cancer cell line: an in vitro study. | <https://doi.org/10.1007/s11033-023-08828-0> | Combination therapy |
| **23** | Network pharmacology to explore the molecular mechanisms of Prunella vulgaris for treating thyroid cancer. | <https://doi.org/10.1097/MD.0000000000034871> | Irrelevant outcome |
| **24** | Neuroprotective Assessment of Nutraceutical (Betanin) in Neuroblastoma Cell Line SHSY-5Y: An in-Vitro and in-Silico Approach. | <https://doi.org/10.1007/s11064-024-04312-8> | In silico study design present |
| **25** | Design, Synthesis and Gene Modulation Insights into Pigments Derived from Tryptophan-Betaxanthin, Which Act against Tumor Development in Caenorhabditis elegans. | <https://doi.org/10.3390/ijms25010063> | In silico study design present |
| **26** | Predictable regulation of gut microbiome in immunotherapeutic efficacy of gastric cancer. | <https://doi.org/10.1038/s41435-024-00306-2> | Wrong intervention |
| **27** | Redox Properties, Bioactivity and Health Effects of Indicaxanthin, a Bioavailable Phytochemical from Opuntia ficus indica, L.: A Critical Review of Accumulated Evidence and Perspectives. | <https://doi.org/10.3390/antiox11122364> | Review |
| **28** | Phytochemicals in quinoa and amaranth grains and their antioxidant, anti-inflammatory, and potential health beneficial effects: a review. | <https://doi.org/10.1002/mnfr.201600767> | Review |
| **29** | Neuroprotective effects of betanin in mice with cerebral ischemia-reperfusion injury. | <https://doi.org/10.1538/expanim.22-0176> | Other disease |
| **30** | In Vitro Screening and Lipid-Lowering Effect of Prickly Pear (Opuntia Ficus-Indica L. Mill.) Fruit Extracts in 3T3-L1 Pre-Adipocytes and Mature Adipocytes. | <https://doi.org/10.1007/s11130-023-01137-8> | Other disease |
| **31** | The Profile of Phenolic Compounds Identified in Pitaya Fruits, Health Effects, and Food Applications: An Integrative Review. | <https://doi.org/10.3390/plants13213020> | Review |
| **32** | C-Glycosyl Flavonoids from Beta vulgaris Cicla and Betalains from Beta vulgaris rubra: Antioxidant, Anticancer and Antiinflammatory Activities-A Review. | <https://doi.org/10.1002/ptr.5819> | Review |
| **33** | Pitahaya Peel: A By-Product with Great Phytochemical Potential, Biological Activity, and Functional Application. | <https://doi.org/10.3390/molecules27165339> | Review |
| **34** | Beetroot and leaf extracts present protective effects against prostate cancer cells, inhibiting cell proliferation, migration, and growth signaling pathways. | <https://doi.org/10.1002/ptr.7197> | Wrong intervention |
| **35** | Targeting FGL2, a molecular drug target for glioblastoma, with natural compounds through virtual screening method. | <https://doi.org/10.4155/fmc-2020-0331> | In silico study design present |
| **36** | Delivery systems for betalains: from stabilization strategies to precision nutrition applications. | <https://doi.org/10.1016/j.foodres.2025.118095> | In silico study design present |
| **37** | Fruit Juices of Etcho (Pachycereus pecten-aboriginum) and Giant Cardon (Pachycereus pringlei) are Sources of Health-Promoting Ingredients with Potential Anticancer Properties. | <https://doi.org/10.1007/s11130-023-01099-x> | Irrelevant outcome |
| **38** | Eco-friendly synthesis of betanin-conjugated zinc oxide nanoparticles: antimicrobial efficacy and apoptotic pathway activation in oral cancer cells. | <https://doi.org/10.1007/s11033-024-10039-0> | In silico study design present |
| **39** | Antioxidant Activity and Anticarcinogenic Effect of Extracts from Bouvardia ternifolia (Cav.) Schltdl. | <https://doi.org/10.3390/life13122319> | Other disease |
| **40** | Beetroot peel flour: Characterization, betalains profile, in silico ADMET properties and in vitro biological activity. | <https://doi.org/10.1016/j.foodchem.2025.143402> | In silico study design present |
| **41** | A novel smart PEGylated gelatin nanoparticle for co-delivery of doxorubicin and betanin: A strategy for enhancing the therapeutic efficacy of chemotherapy. | <https://doi.org/10.1016/j.msec.2018.12.104> | Combination therapy |
| **42** | Exploring anticancer potential of betanin in DMBA-induced oral squamous cell carcinoma: an in silico and experimental study. | <https://doi.org/10.1007/s00210-025-03909-2> | In silico study design present |
| **43** | DNA damage and apoptosis in blood neutrophils of inflammatory bowel disease patients and in Caco-2 cells in vitro exposed to betanin. | <https://doi.org/10.5604/17322693.1198989> | Other disease |
| **44** | Betalain exerts cardioprotective and anti-inflammatory effects against the experimental model of heart failure. | <https://doi.org/10.1177/09603271211027933> | Other disease |
| **45** | Betanin Dose-Dependently Ameliorates Allergic Airway Inflammation by Attenuating Th2 Response and Upregulating cAMP-PKA-CREB Pathway in Asthmatic Mice. | <https://doi.org/10.1021/acs.jafc.2c00205> | Other disease |
| **46** | The potential benefits of red beetroot supplementation in health and disease. | <https://doi.org/10.3390/nu7042801> | Review |
| **47** | Betalain Alleviates Airway Inflammation in an Ovalbumin-Induced-Asthma Mouse Model via the TGF-β1/Smad Signaling Pathway. | <https://doi.org/10.1615/JEnvironPatholToxicolOncol.2021037050> | Other disease |
| **48** | Potential Effects of Bioactive Compounds of Plant-Based Foods and Medicinal Plants in Chronic Kidney Disease and Dialysis: A Systematic Review. | <https://doi.org/10.3390/nu16244321> | Irrelevant outcome |
| **49** | Betalains: colours for human health. | <https://doi.org/10.1080/14786419.2022.2106481> | Irrelevant outcome |
| **50** | Betalains, the nature-inspired pigments, in health and diseases. | <https://doi.org/10.1080/10408398.2018.1479830> | Review |
| **51** | Bioactive compounds and health benefits of Pereskioideae and Cactoideae: A review. | <https://doi.org/10.1016/j.foodchem.2020.126961> | Review |
| **52** | Positive impact of indicaxanthin from Opuntia ficus-indica fruit on high-fat diet-induced neuronal damage and gut microbiota dysbiosis. | <https://doi.org/10.4103/NRR.NRR-D-23-02039> | Review |
| **53** | Betanin Mitigates Inflammation and Ankle Joint Damage by Inhibiting the MAPK/NF-κB Pathway in Arthritis Triggered by Type II Collagen in Rats. | <https://doi.org/10.2174/0113862073344449241122064531> | Other disease |
| **54** | Protective Effects of Betanin in Acute and Subacute Periods in Experimental Colitis Induced by Trinitrobenzene Sulfonic Acid. | <https://doi.org/10.3390/nu18010086> | Other disease |
| **55** | A preclinical study on effect of betanin on sodium fluoride induced hepatorenal toxicity in Wistar rats. | <https://doi.org/10.1515/jcim-2024-0262> | Other disease |
| **56** | DNA damage and apoptosis in blood neutrophils of inflammatory bowel disease patients and in Caco-2 cells in vitro exposed to betanin. | <https://doi.org/10.5604/17322693.1198989> | Other disease |
| **57** | The interplay of TapSAKI and NEAT-1 as potential modulators in gentamicin-induced acute kidney injury via orchestrating miR-22-3p/TLR4/MyD88/NF-κB/IL-1 β milieu: Novel therapeutic approach of Betanin. | <https://doi.org/10.1016/j.intimp.2024.113577> | Other disease |
| **58** | Betanin attenuates oxidative stress and inflammatory reaction in kidney of paraquat-treated rat. | <https://doi.org/10.1016/j.fct.2015.01.018> | Other disease |
| **59** | Ameliorative effect of betanin on experimental cisplatin-induced liver injury; the novel impact of miRNA-34a on the SIRT1/PGC-1α signaling pathway. | <https://doi.org/10.1002/jbt.22753> | Other disease |
| **60** | The Responses of Bioactive Betanin Pigment and Its Derivatives from a Red Beetroot (Beta vulgaris L.) Betalain-Rich Extract to Hypochlorous Acid. | <https://doi.org/10.3390/ijms22031155> | In silico study design present |
| **61** | Nutraceutical Potential of Djulis (Chenopodium formosanum) Hull: Phytochemicals, Antioxidant Activity, and Liver Protection. | <https://doi.org/10.3390/antiox13060721> | Other disease |
| **62** | Synergistic protective effect of Beta vulgaris with meso-2,3-dimercaptosuccinic acid against lead-induced neurotoxicity in male rats. | <https://doi.org/10.1038/s41598-020-80669-4> | Other disease |
| **63** | Beetroot and Sodium Nitrate Ameliorate Cardiometabolic Changes in Diet-Induced Obese Hypertensive Rats. | <https://doi.org/10.1002/mnfr.201700478> | Other disease |
| **64** | Betanin ameliorates lipopolysaccharide-caused testis damage via multiple signal networks. | <https://doi.org/10.1080/09603123.2025.2474701> | Other disease |
| **65** | Reduction of doxorubicin-induced cytotoxicity and mitochondrial damage by betanin in rat isolated cardiomyocytes and mitochondria. | <https://doi.org/10.1177/09603271211022800> | Other disease |
| **66** | Robust W/O/W Emulsion Stabilized by Genipin-Cross-Linked Sugar Beet Pectin-Bovine Serum Albumin Nanoparticles: Co-encapsulation of Betanin and Curcumin. | <https://doi.org/10.1021/acs.jafc.0c05212> | Combination therapy |
| **67** | Stimulation of autophagy prevents intestinal mucosal inflammation and ameliorates murine colitis. | <https://doi.org/10.1111/bph.13860> | Wrong intervention |
| **68** | Minimal muscle damage after a marathon and no influence of beetroot juice on inflammation and recovery. | <https://doi.org/10.1139/apnm-2016-0525> | Wrong population |
| **69** | Impact of Red Beetroot Juice on Vascular Endothelial Function and Cardiometabolic Responses to a High-Fat Meal in Middle-Aged/Older Adults with Overweight and Obesity: A Randomized, Double-Blind, Placebo-Controlled, Crossover Trial. | <https://doi.org/10.1093/cdn/nzz113> | Wrong population |
| **70** | A randomized clinical trial of beetroot juice consumption on inflammatory markers and oxidative stress in patients with type 2 diabetes. | <https://doi.org/10.1111/1750-3841.16365> | Wrong population |
| **71** | The effects of beetroot juice supplementation on indices of muscle damage following eccentric exercise. | <https://doi.org/10.1007/s00421-015-3290-x> | Wrong population |
| **72** | Beetroot juice intake positively influenced gut microbiota and inflammation but failed to improve functional outcomes in adults with long COVID: A pilot randomized controlled trial. | <https://doi.org/10.1016/j.clnu.2024.11.023> | Wrong population |
| **73** | Betalains Alleviate Exercise-Induced Oxidative Stress, Inflammation, and Fatigue and Improve Sports Performance: an Update on Recent Advancements. | <https://doi.org/10.1007/s13668-023-00500-0> | Wrong population |
| **74** | Effect of 1-week betalain-rich beetroot concentrate supplementation on cycling performance and select physiological parameters. | <https://doi.org/10.1007/s00421-018-3973-1> | Wrong population |
| **75** | The Phytochemical Indicaxanthin Synergistically Enhances Cisplatin-Induced Apoptosis in HeLa Cells via Oxidative Stress-Dependent p53/p21^waf1^ Axis | <https://doi.org/10.3390/biom10070994> | Combination therapy |
| **76** | Indicaxanthin Induces Autophagy in Intestinal Epithelial Cancer Cells by Epigenetic Mechanisms Involving DNA Methylation | <https://doi.org/10.3390/nu15153495> | In silico |

**Supplementary Appendix S2:** Risk of Bias Assessment for in vivo studies using the SYRCLE Tool (n = 09).

| **Study** | **Randomization** | **Baseline** | **Performance bias** | **Detection bias** | **Attrition** | **Reporting bias** | **Other bias** |
| --- | --- | --- | --- | --- | --- | --- | --- |
| [13] | Unclear | Unclear | High | High | Low | Unclear | Unclear |
| [38] | Unclear | Low | High | High | Unclear | Unclear | Low |
| [39] | Unclear | Unclear | High | Unclear | Low | Unclear | Low |
| [28] | Unclear | Unclear | High | Unclear | Unclear | Unclear | Unclear |
| [29] | Low | Unclear | High | Unclear | Low | Low | Low |
| [40] | Unclear | Unclear | High | Unclear | Low | Low | Low |
| [41] | Low | Low | High | High | Low | Low | Low |
| [42] | High | Low | High | High | Unclear | Unclear | Low |
| [43] | Low | High | Unclear | High | High | Unclear | Low |

**Supplementary Appendix S3:** Domains related to the SYRCLE Tool.

1. Selection Bias (Randomization)

Was the allocation of animals to experimental and control groups randomized?

2. Baseline Characteristics

Were the groups similar at baseline in terms of key characteristics (e.g., tumour size, weight, inflammation status)?

3. Performance Bias (Blinding of Caregivers/Researchers)

Were the caregivers or investigators blinded to the group allocation during the study?

4. Detection Bias (Blinding of Outcome Assessment)

Was the outcome assessment (e.g., tumor growth, cytokine levels, histopathology) blinded to treatment allocation?

5. Attrition Bias (Incomplete Outcome Data)

Were incomplete data (e.g., excluded animals, deaths) adequately addressed and explained?

6. Reporting Bias (Selective Reporting)

Were all pre-specified outcomes reported, and not only significant or favorable results?

7. Other Bias (Confounding/Conflict of Interest)

Were potential sources of bias, such as housing, funding, or conflicts of interest, addressed?

**Supplementary Table S1:** PRISMA checklist of included studies**.**

| **Section and Topic** | **Item #** | **Checklist item** | **Location where item is reported** |
| --- | --- | --- | --- |
| **TITLE** | | |  |
| Title | 1 | Title page: Anticancer and Anti-inflammatory Potential of Betalains: A Systematic Review on Preclinical Studies | 1 |
| **ABSTRACT** | | |  |
| Abstract | 2 | See the PRISMA 2020 for Abstracts checklist. | 1 |
| **INTRODUCTION** | | |  |
| Rationale | 3 | Introduction (first two paragraphs, background on cancer and inflammation, and rationale for betalain) | 1, 2 |
| Objectives | 4 | Introduction (last paragraph, hypothesis, and aims) | 2 |
| **METHODS** | | |  |
| Eligibility criteria | 5 | Methods → Eligibility criteria | 4, 5 |
| Information sources | 6 | Methods → Search strategy (databases: PubMed, ScienceDirect, Wiley Online Library, and Taylor & Francis; date: June 02, 2025) | 4 |
| Search strategy | 7 | Methods → Search strategy (full string provided) | 4 |
| Selection process | 8 | Methods → Study selection process (PRISMA flow) | 4, 12 |
| Data collection process | 9 | Methods → Data extraction (PRISMA framework, PICOS) | 6 |
| Data items | 10a | Methods → Data extraction (anticancer activity, anti-inflammatory markers) | 6 |
|  | 10b | Methods → Data extraction (population, intervention, comparator, study design) | 3, 4, 6 |
| Study risk of bias assessment | 11 | Methods → Risk of Bias Assessment (SYRCLE tool, customized RoB tool, QUIN tool) | 5, 6 |
| Effect measures | 12 | Methods → Data Synthesis and Analysis | Not applicable |
| Synthesis methods | 13a | Methods → Data Synthesis, Subgroup Analysis, Meta-regression | Not applicable |
|  | 13b | Methods → Data Synthesis, Subgroup Analysis, Meta-regression | Not applicable |
|  | 13c | Methods → Data Synthesis, Subgroup Analysis, Meta-regression | Not applicable |
|  | 13d | Methods → Data Synthesis, Subgroup Analysis, Meta-regression | Not applicable |
|  | 13e | Methods → Data Synthesis, Subgroup Analysis, Meta-regression | Not applicable |
|  | 13f | Methods → Data Synthesis, Subgroup Analysis, Meta-regression | Not applicable |
| Reporting bias assessment | 14 | Methods → Reporting Bias (summary plot, heatmap, traffic plot) | 5 to 6, 14 to 15 |
| Certainty assessment | 15 | Methods → Risk of Bias + Discussion limitations | 5, 6, 23 |
| **RESULTS** | | |  |
| Study selection | 16a | Results → Literature Search + Figure 2 (flow diagram) | 5, 12 |
|  | 16b | Results → Literature Search (40 excluded: studies with other compounds, other study designs) | 5, 12 |
| Study characteristics | 17 | Results → Study Characteristics + Table 1 and 2 | 6 to 11, 13 |
| Risk of bias in studies | 18 | Results → Risk of Bias (Figures 3 to 6) | 14, 15 |
| Results of individual studies | 19 | Results → Tables 1 and 2 (study design, key outcome) | 6 to 11 |
| Results of syntheses | 20a | Results → Meta-analysis, subgroup, sensitivity, heterogeneity | Not applicable |
|  | 20b | Results → Meta-analysis, subgroup, sensitivity, heterogeneity | Not applicable |
|  | 20c | Results → Meta-analysis, subgroup, sensitivity, heterogeneity | Not applicable |
|  | 20d | Results → Meta-analysis, subgroup, sensitivity, heterogeneity | Not applicable |
| Reporting biases | 21 | Results → Publication Bias (summary plot, heatmap, traffic plot) | 13 to 15 |
| Certainty of evidence | 22 | Results + Discussion (limitations, heterogeneity, robustness) | 15 to 19, 23 |
| **DISCUSSION** | | |  |
| Discussion | 23a | Discussion (overview of findings) | 19 to 23 |
|  | 23b | Discussion (limitations: heterogeneity, lack of clinical data) | 23 |
|  | 23c | Discussion (bias, heterogeneity, publication bias) | 23 |
|  | 23d | Discussion (future studies, clinical applications) | 23, 24 |
| **OTHER INFORMATION** | | |  |
| Registration and protocol | 24a | Methods → PROSPERO submission ID 1172411 | 3 |
|  | 24b | Methods → PROSPERO submission ID 1172411 | 3 |
|  | 24c | Methods → PROSPERO submission ID 1172411 | 3 |
| Support | 25 | Declarations → Funding: none | 24 |
| Competing interests | 26 | Declarations → No conflicts | 25 |
| Availability of data, code and other materials | 27 | Declarations → Available upon request | 25 |

**Supplementary Table S2:** Risk of bias assessment of in vitro studies using a customized tool (n=12).

| - Sr. | - Author/ Year/ Country | - 1. Were the cancer cell lines used reported? | - 2. Was the duration of exposure to the cancer cell line reported? | - 3. Was the concentration of the cancer cell culture employed reported? | - 4. Was a standard culture medium used for treatment control? | - 5. Were reliable tools used to assess the outcome? | - 6. Were the experiments conducted in triplicate? | - 7. Was more than one independent experiment performed? |
| --- | --- | --- | --- | --- | --- | --- | --- | --- |
| - 1. | - Zou et al., 2022 - (China) | - Low | - Low | - Unclear | - Low | - Low | - Low | - Unclear |
| - 2. | - Saber et al., 2023 - (Iran) | - Low | - Low | - Low | - Low | - Low | - Low | - Low |
| - 3. | - Shafreen and Kumar, 2022 - (India) | - Low | - Low | - Low | - Low | - Low | - Low | - Unclear |
| - 4. | - Liu et al., 2023 - (China) | - Low | - Low | - Low | - Low | - Low | - Low | - Low |
| - 5. | - Highwacki et al., 2015 - (France) | - Low | - Low | - Low | - Low | - Low | - Low | - Low |
| - 6. | - Chandrasekaran et al., 2022 - (India) | - Low | - Low | - Low | - Low | - Low | - Low | - Unclear |
| - 7. | - Scarpa et al., 2016 - (Italy) | - Low | - Low | - Low | - Low | - Low | - Low | - Low |
| - **8.** | - Zand et al., 2024 - (Hungary) | - Low | - Low | - Low | - Low | - Low | - Low | - Unclear |
| - 9. | - Hirad et al., 2025 - (Saudi Arabia) | - Low | - Low | - Low | - Unclear | - Low | - Low | - Unclear |
| - 10. | - Farabegoli et al., 2017 - (Italy) | - Low | - Low | - Unclear | - Low | - Low | - Low | - Low |
| - 11. | - Yin et al., 2021 - (China) | - Low | - Low | - Low | - Low | - Low | - Low | - Low |
| - 12. | - Salimi et al., 2021 (Iran) | - Low | - Low | - High | - Low | - Low | - Low | - Low |

**Supplementary Table S3.1:** Risk of Bias Assessment of inflammation-related *in vitro* studies using the QUIN Tool (n=05).

| **Author-Year** | **Study Aim & Rationale** | **Test Item (Betalains)** | **Test System (Cells)** | **Study Design** | **Outcome Assessment** | **Data Analysis** | **Documentation & Transparency** | **Funding / Conflict of Interest** | **Overall Risk** |
| --- | --- | --- | --- | --- | --- | --- | --- | --- | --- |
| Fernando et al., 2023 | Low | Low | Low | Low | Low | Low | Low | Low | Low |
| Wang et al., 2022 | Low | Low | Unclear | Unclear | Low | Low | Low | Low | Low |
| Smeriglio et al., 2021 | High | Low | Unclear | Unclear | Low | Low | Unclear | Low | High |
| Rusak et al., 2024 | Low | Low | Low | Low | Low | Low | Low | High | Low |
| Kumorkiewicz-Jamroet al., 2025 | Low | Low | Low | Low | Low | Low | Low | Low | Low |

**Supplementary Table S3.2:** Domains related to the QUIN Tool.

| **Domain** | **Key Question per domain** |
| --- | --- |
| 1. Study Aim & Rationale | \| Is the study's aim or hypothesis clearly stated and scientifically justified? \|  \|  \| \| --- \| --- \| --- \| |
| 2. Test Item (Betalains) | Is the betalain compound or extract clearly described (identity, purity, source, preparation)? |
| 3. Test System (Cells) | Is the cell line or primary culture clearly identified, authenticated, and appropriate for the study aim? |
| 4. Study Design | Were relevant controls included (negative, vehicle, positive), and was randomization/blinding applied where feasible? |
| 5. Outcome Assessment | Are the outcomes (e.g., IL-6, TNF-α, AMPK) measured with validated methods and reported transparently? |
| 6. Data Analysis | Were biological/technical replicates adequate, and were statistical methods appropriate and clearly described? |
| 7. Documentation & Transparency | Is the methodology described in enough detail to allow reproducibility by others? |
| 8. Funding / Conflict of Interest | Were funding sources and potential conflicts of interest reported transparently? |

|  |  |
| --- | --- |
